# Targeting lactate dehydrogenase B-dependent mitochondrial metabolism affects tumor initiating cells and inhibits tumorigenesis of non-small cell lung cancer by inducing mtDNA damage

**DOI:** 10.1007/s00018-022-04453-5

**Published:** 2022-07-25

**Authors:** Haibin Deng, Yanyun Gao, Verdiana Trappetti, Damian Hertig, Darya Karatkevich, Tereza Losmanova, Christian Urzi, Huixiang Ge, Gerrit Adriaan Geest, Remy Bruggmann, Valentin Djonov, Jean-Marc Nuoffer, Peter Vermathen, Nicola Zamboni, Carsten Riether, Adrian Ochsenbein, Ren-Wang Peng, Gregor Jan Kocher, Ralph Alexander Schmid, Patrick Dorn, Thomas Michael Marti

**Affiliations:** 1 Department of General Thoracic Surgery, Inselspital, Bern University Hospital, Bern, Switzerland; 2 Department for BioMedical Research, University of Bern, Bern, Switzerland; 3 Institute of Anatomy, University of Bern, Bern, Switzerland; 4 Department of Neuroradiology, University of Bern, Bern, Switzerland; 5 Institute of Clinical Chemistry, University Hospital Bern, Bern, Switzerland; 6 Graduate School of Cellular and Biomedical Sciences, University of Bern, Bern, Switzerland; 7 Institute of Pathology, University of Bern, Bern, Switzerland; 8 Interfaculty Bioinformatics Unit, Swiss Institute of Bioinformatics, University of Bern, Bern, Switzerland; 9 Department of Pediatric Endocrinology, Diabetology and Metabolism, University Children’s Hospital of Bern, Bern, Switzerland; 10 Translational Imaging Center (TIC), Swiss Institute for Translational and Entrepreneurial Medicine, Bern, Switzerland; 11 Institute for Molecular Systems Biology, ETH Zurich, Zurich, Switzerland; 12 Department of Medical Oncology, Inselspital, Bern University Hospital, Bern, Switzerland

**Keywords:** Lung cancer, Cancer stem cells, Tumorigenicity, Mitochondrial metabolism, Cellular plasticity, Mitochondrial DNA, Nucleotide metabolism

## Abstract

Once considered a waste product of anaerobic cellular metabolism, lactate has been identified as a critical regulator of tumorigenesis, maintenance, and progression. The putative primary function of lactate dehydrogenase B (LDHB) is to catalyze the conversion of lactate to pyruvate; however, its role in regulating metabolism during tumorigenesis is largely unknown. To determine whether LDHB plays a pivotal role in tumorigenesis, we performed 2D and 3D in vitro experiments, utilized a conventional xenograft tumor model, and developed a novel genetically engineered mouse model (GEMM) of non-small cell lung cancer (NSCLC), in which we combined an *LDHB* deletion allele with an inducible model of lung adenocarcinoma driven by the concomitant loss of *p53* (also known as Trp53) and expression of oncogenic *KRAS* (G12D) (KP). Here, we show that epithelial-like, tumor-initiating NSCLC cells feature oxidative phosphorylation (OXPHOS) phenotype that is regulated by LDHB-mediated lactate metabolism. We show that silencing of *LDHB* induces persistent mitochondrial DNA damage, decreases mitochondrial respiratory complex activity and OXPHOS, resulting in reduced levels of mitochondria-dependent metabolites, e.g., TCA intermediates, amino acids, and nucleotides. Inhibition of LDHB dramatically reduced the survival of tumor-initiating cells and sphere formation in vitro, which can be partially restored by nucleotide supplementation. In addition, *LDHB* silencing reduced tumor initiation and growth of xenograft tumors. Furthermore, we report for the first time that homozygous deletion of *LDHB* significantly reduced lung tumorigenesis upon the concomitant loss of *Tp53* and expression of oncogenic *KRAS* without considerably affecting the animal’s health status, thereby identifying LDHB as a potential target for NSCLC therapy. In conclusion, our study shows for the first time that LDHB is essential for the maintenance of mitochondrial metabolism, especially nucleotide metabolism, demonstrating that LDHB is crucial for the survival and proliferation of NSCLC tumor-initiating cells and tumorigenesis.

**Supplementary Information:**

The online version contains supplementary material available at 10.1007/s00018-022-04453-5.

## Background

The tumor is a complex system containing a subpopulation of highly tumorigenic cells referred to as cancer stem cells or tumor-initiating cells (TICs) [1]. TICs have been discovered in many solid tumors, including breast, brain, skin, lung, pancreatic, and colon [2–7]. TICs are thought to contribute to the tumor initiation, maintenance, progression, resistance to treatments, and recurrence or metastasis of cancer [8–11]. In lung cancer, TICs have been identified using several markers, e.g., drug-resistant side-population, CD133^+^, ALDH^high^, and EpCAM^+^ [12–14]. Recently, glycine decarboxylase (GLDC) was identified as the most robust TIC marker in NSCLC [12]. Intriguingly, GLDC is mainly expressed in the mitochondria [15, 16]. A high level of oxidative phosphorylation (OPXHOS) correlates with increased sphere formation and tumor growth capacity in NSCLC [17]. In addition, a recent study has shown that a functional OXPHOS is critical for tumorigenesis. Specifically, loss of mitochondria-dependent pyrimidine synthesis rather than mitochondria-dependent ATP production is responsible for the loss of tumorigenic ability [18]. Indeed, loss of mitochondria-dependent nucleotide synthesis is synthetically lethal in cancers induced by a KRAS mutation [16]. Although considered a waste product of anaerobic glycolysis (e.g., Warburg effect), lactate has been shown to serve as a source of respiration in cancer, and targeting lactate-fueled respiration can selectively kill tumor cells in mice [19]. More recently, research revealed that lactate is the primary carbon source for the TCA cycle in vivo, providing substrate and electrons for oxidative phosphorylation (OXPHOS) in normal tissue and lung tumors [20, 21]. Thus, lactate drives mitochondrial metabolism, but the relationship between lactate and survival of NSCLC TICs cancer is not clear.

The tetrameric enzyme lactate dehydrogenase (LDH), encoded by the genes lactate dehydrogenase A and B (*LDHA* and *LDHB*, respectively), catalyzes the interconversion of pyruvate and lactate using NADH/NAD^+^ as a co-substrate (reviewed in Ref. [22]). LDHA, particularly the LDHA-homo-tetramer, converts pyruvate to lactate, whereas LDHB primarily converts lactate to pyruvate. Only the double-knockout of *LDHA* and *LDHB* entirely suppressed LDH activity and lactate secretion [23]. Thus, LDHB may at least partially replace the function of LDHA in promoting glycolysis. LDHA has been shown to be essential for tumorigenesis, growth, and progression of different cancers (reviewed in Refs. [22, 24, 25]). Furthermore, LDHA abrogation has been shown to reduce tumorigenesis and tumor growth and decrease the survival and proliferation of TICs in a novel inducible NSCLC mouse models in the context of *KRAS* or *EGFR* mutations [26]. Interestingly, abrogation of LDHA results not only in inhibition of glycolysis but also in metabolic reprogramming leading to enhanced mitochondrial metabolism [26], suggesting that LDHA expression is essential for glycolysis but may not be critical for maintaining mitochondrial function. However, LDHB is localized to the mitochondria and treatment with an LDH inhibitor decreased respiration of isolated mitochondria cultured in lactate but not in pyruvate [27]. Taken together, LDHB may be more related to mitochondrial metabolism and its inhibition may even have an opposite effect on mitochondrial metabolism compared to LDHA. The relationship between LDHB expression and cancer is complex: LDHB is silenced by promoter methylation in several cancers, but increased LDHB expression has been described in several adenocarcinomas, including NSCLC [28]. Increased LDHB expression is associated with poor survival in many cancers [29, 30]. In particular, LDHB has been shown to be required for the growth of *KRAS*-mutated lung cancer cells in vitro and in vivo [31] and to play a critical role in hyperactive mTOR-mediated tumorigenesis [32]. However, whether TICs of NSCLC rely on high levels of OXPHOS, whether LDHB is essential for lactate-mediated OXPHOS for TICs, and especially how LDHB regulates mitochondrial metabolism, remains largely unknown.

Our current study demonstrated that TICs in NSCLC are characterized by an OXPHOS phenotype, which relies on LDHB activity. Inhibition of LDHB induces persistence of mtDNA damage and dramatically reduces OXPHOS and mitochondrial metabolism-dependent nucleotide metabolism leading to a reduction in survival of TICs in NSCLC. Our study revealed that LDHB has a vital role in tumorigenesis, both mouse xenograft tumor models and GEMMs. Thus, our research revealed that LDHB is essential to maintain mitochondrial DNA integrity and mitochondrial metabolism, which is crucial for tumor-initiating cells and tumorigenesis.

## Results

### TICs are characterized as OXPHOS phenotype and tend to lactate utilization

**Fig. 1:**
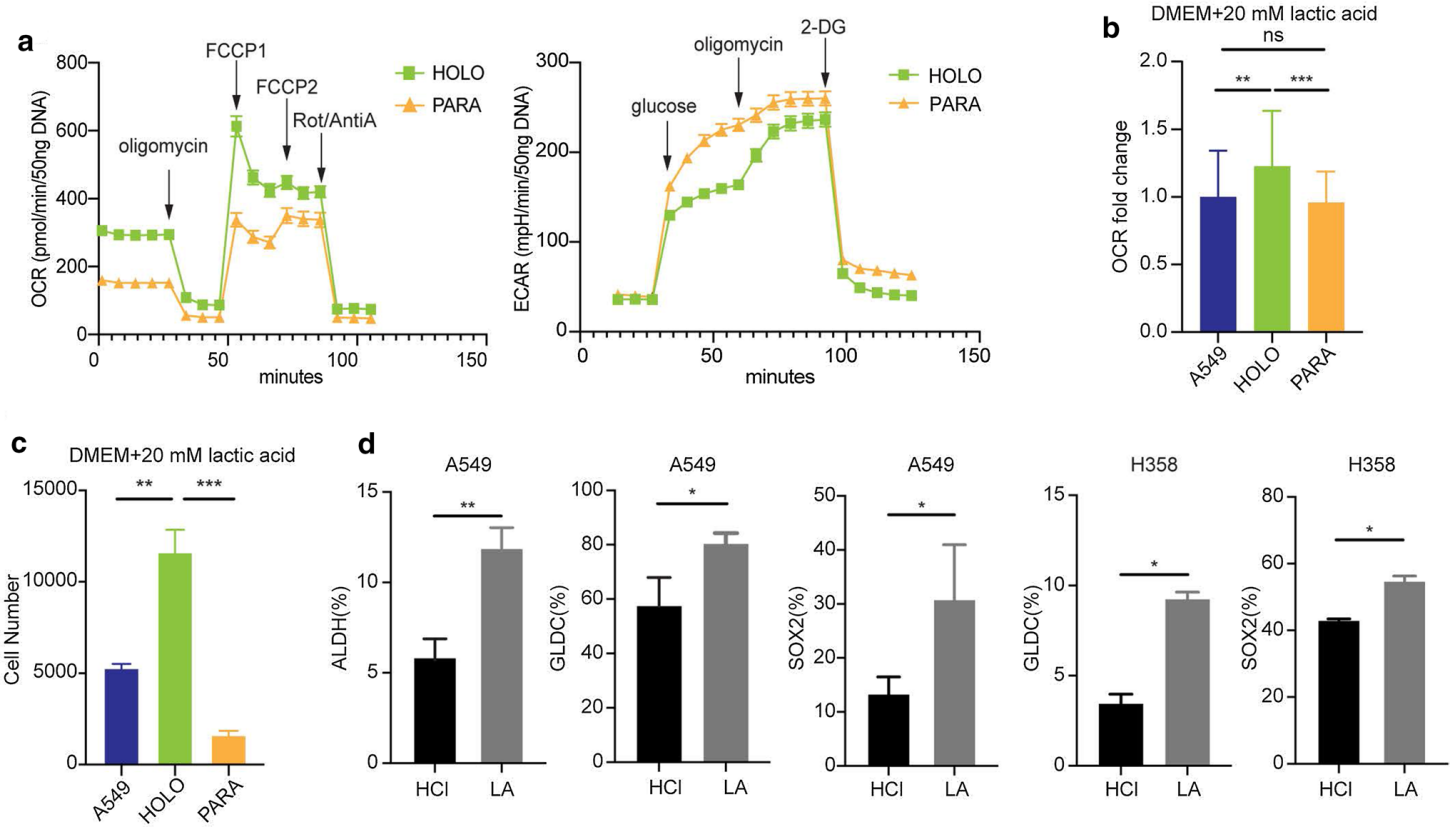
TICs are characterized as OXPHOS phenotype and tend to lactate utilization. **a** Representative plot showing mean ± SEM of the real-time oxygen consumption rate (OCR) from Mito Stress Test and extracellular acidification rate (ECAR) from Glycolysis Stress Test of holoclonal (HOLO) and paraclonal (PARA) cells was measured in 96-well plates cultured in DMEM medium using the Seahorse XFe96 Analyzer. For Mito Stress Test, 1 μM oligomycin, 1.0 μM and 1.5 μM FCCP, a mixture of 1 μM rotenone and 1 μM antimycin A were added sequentially (*n* = 24 technical replicates with 3–5 readings). For Glycolysis Stress Test, 10 mM glucose, 1 μM oligomycin, 50 mM 2-DG were added sequentially (*n* = 22–23 technical replicates with 3–5 readings). **b** A549, HOLO, and PARA cells were pretreated in DMEM medium (no glucose, no glutamine, no pyruvate) with 20 mM lactic acid for 24 h and adjusted to a pH of 6.8, then OCR was measured as described above. All basal OCR was normalized to mean basal OCR of A549 cells. The data was presented as OCR fold change to A549 cell line. The error bars represent mean ± SD (*n* = 4 biological replicates). *ns* no significant difference, **P < 0.01, ***P < 0.001 (Ordinary one-way ANOVA). **c** A549, HOLO, and PARA cells were seeded in 6-well plates and cultured overnight in DMEM/F12 medium. Cells were then changed into starvation DMEM medium (no glucose, no glutamine, no pyruvate) containing 20 mM L-lactic acid and adjusted to pH 6.8. Cell number was determined after 6 days (*n* = 3). **P < 0.01, ***P < 0.001 (Ordinary one-way ANOVA). **d** A549 and H358 cell lines were cultured in 100 mm × 20 mm dishes containing DMEM/F12 medium to equilibrate overnight. Cells then were changed into DMEM with 2.5 mM glucose, no pyruvate and no glutamine medium (low-GLC) containing 20 mM L-lactic acid or HCl and adjusted to pH 6.8. After 5 days, cells were harvested and analyzed by FACS by staining with ALDEFLUOR (ALDH) (*n* = 3) or GLDC (*n* = 3–4) or SOX2 (*n* = 3). *P < 0.05, **P < 0.01 (two-tailed paired Student’s *t*-test). Error bars represent mean ± SD

To better understand the metabolic synergy between lung cancer cells, we extended our characterization of subpopulations in the NSCLC cell line A549, where the mesenchymal paraclone subpopulation (PARA) had the lowest tumor-initiating capacity, whereas the epithelial holoclone subpopulation (HOLO) had the highest tumor-initiating capacity [33]. We focused on lactate metabolism, which fuels lung cancer metabolism [21]. Reanalysis of our previously published data [33] revealed that hexokinase 2 (HK2), LDHA, and the sodium lactate transporter MCT4 (encoded by SLC16A3) catalyzing the first step of glucose metabolism, lactate synthesis, and lactate secretion, respectively, are significantly overexpressed in PARA compared to the HOLO (Supplementary Fig. S1a). In contrast, LDHB, which mainly converts lactate and NAD^+^ to pyruvate and NADH, is expressed at higher levels in HOLO (Supplementary Fig. S1a). Therefore, we postulated a simple model in which A549 paraclonal cells process glucose by glycolysis and secrete lactate, which drives energy metabolism in holoclonal cells. In agreement with this model, the basal oxygen consumption rate (OCR) of paraclonal A549 cells is decreased compared to holoclonal cells (Fig. 1a and Supplementary Fig. S1b). In contrast, the glucose-dependent extracellular acidification rate (ECAR), which is associated with glycolysis, is increased in paraclonal A549 cells (Fig. 1a). Compared to paraclonal cells, the decrease in basal OCR of holoclonal cells was significantly more pronounced after treatment with oligomycin, which is an inhibitor of ATP synthase, indicating that the ATP production in holoclonal cells is more dependent on OXPHOS compared to paraclonal cells (Fig. 1a and Supplementary Fig. S1c). Indeed, the hallmarks of functional OXPHOS, e.g., maximal respiration and spare respiration, were also significantly increased in holoclonal A549 cells (Fig. 1a and Supplementary Fig. S1c). Interestingly, although glycolysis and glycolytic capacity were increased compared with holoclonal cells, the glycolytic reserve was negligible in paraclonal cells, suggesting that glycolysis in paraclonal cells runs at nearly full capacity (Supplementary Fig. S1d).

Moreover, lactate supplementation specifically increased the OCR of holoclonal cells (Fig. 1b) and rescued the survival of holoclonal cells under glucose deprivation (Fig. 1c). Consistent with this, lactate supplementation also increased in the A549 and H358 cell lines the proportion of the subpopulations characterized by elevated GLDC, Aldehyde dehydrogenase (ALDH) activity and SOX2 expression, critical markers associated with heightened tumor initiation capacity [12, 34] (Fig. 1d and Supplementary Fig. S1e).

In summary, our experiments support our simple model of metabolic symbiosis in which A549 paraclonal cells exhibit increased glycolysis, as evidenced by increased ECAR and expression of glycolysis-associated genes, and secrete lactate, which drives the tumor-initiating phenotype of holoclonal cells associated with increased OXPHOS, ALDH activity, SOX2 expression, and GLDC expression.

### LDHB activity is critical for the maintenance of OXPHOS

**Fig. 2:**
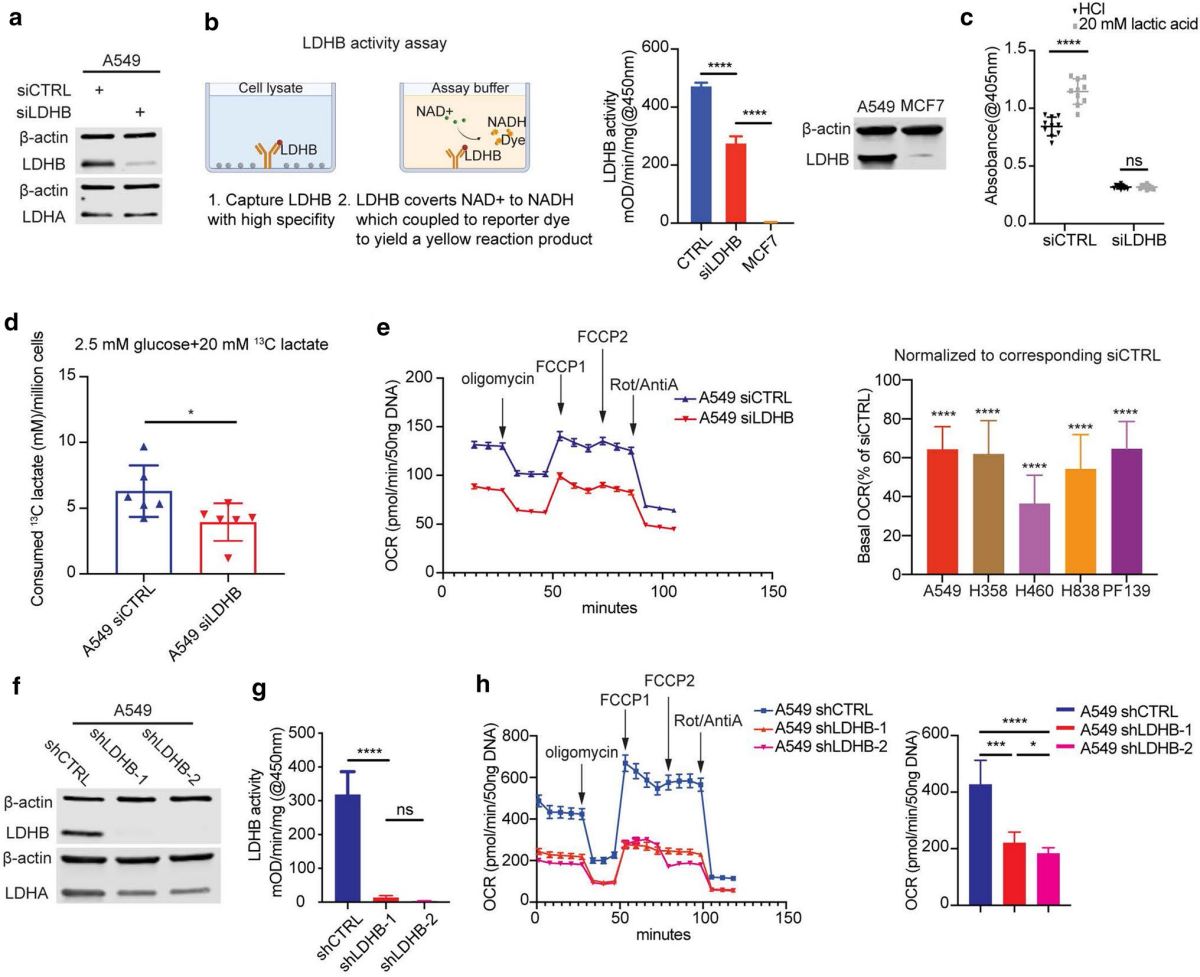
LDHB mediated lactate utilization is critical to OXPHOS. **a** Immunoblot analysis of A549 cells transfected with control siRNA (siCTRL) or LDHB-specific siRNA (siLDHB) (10 nM) using Lipofectamine 2000 after 48 h. β-actin was used as the loading control. **b** LDHB activity levels were determined in A549 cells 48 h after transfection with siCTRL or siLDHB using an enzymatic colorimetric kit. The MCF7 cell line, expressing LDHA but not LDHB protein, was used as a negative control. Mean OD values were normalized to the number of cell lysates and reaction time. ****P < 0.0001 (Ordinary one-way ANOVA). The LDHB protein expression of MCF7 was analysis by western blot. **c** siCTRL and siLDHB cells were plated at 3000 cells/well in 96-well plates for overnight culture. Cells then were changed into DMEM with 2.5 mM glucose, no pyruvate and no glutamine medium (low-GLC) containing 20 mM L-lactic acid or HCl and adjusted to pH 6.8. After 4 days, cell viability was determined by Acid Phosphatase (APH) Assay. Absorbance was quantified in a Tecan Infinite^®^ M1000 Microplate Reader at 405 nm (*n* = 10). ****P < 0.0001 (Ordinary two-way ANOVA). The error bars represent mean ± SD. **d** siCTRL and siLDHB cells were plated at 1 × 10^5^ cells/well in 6-well plates for overnight culture. Cells then were changed into DMEM medium containing 2.5 mM glucose and 20 mM 3-^13^C sodium L-lactate. After 72 h, supernatants were collected for NMR analysis. The measurement was previously described [36–39]. For each sample, the ^13^C lactate peak of interest was integrated. The absolute integral was then converted to an mM value using the integral of the initial condition with a known concentration of 20 mM lactate. Lactate consumption values were normalized to the corresponding cell number and then used for statistical analysis (*n* = 6). *P < 0.05 (two-tailed unpaired Student’s *t*-test). Data are shown with mean ± SD. **e** Representative plot showing mean ± SEM of the real-time oxygen consumption rate (OCR) of A549 siCTRL and A549 siLDHB cells using the Seahorse XFe96 Analyzer (*n* = 39–40 technical replicates with 3–5 readings). The procedure corresponds to the description in Fig. 1. The basal OCR of cell lines were used to perform the statistical analysis. The normalized OCR were plotted as bar graphs (*n* = 3–4 biological replicates). The error bars represent mean ± SD. ****P < 0.0001 (two-tailed unpaired Student’s *t*-test). **f, g** Immunoblot analysis of A549 shCTRL (scramble control) and shLDHB clones. β-actin was used as a loading control. LDHB activity levels were determined as described above (*n* = 3). The error bars represent mean ± SD. *ns* no significant difference, ****P < 0.0001 (Ordinary one-way ANOVA). **h** Representative plot showing mean ± SEM of the real-time oxygen consumption rate (OCR) of A549 shCTRL and A549 shLDHB cells using the Seahorse XFe96 Analyzer (*n* = 6 technical replicates with 3–5 readings). The basal OCR were plotted as bar graphs (*n* = 4 biological replicates). The error bars represent mean ± SD. *P < 0.05, ***P < 0.001, ****P < 0.0001 (Ordinary one-way ANOVA)

To further dissect how LDHB-mediated lactate metabolism affects NSCLC TICs, we performed a short-term inhibition of LDHB expression using siRNA, which significantly reduced LDHB expression and activity. As a control, MCF7 breast cancer cells, characterized by very low LDHB protein expression levels, exhibited marginal LDHB activity (Fig. 2a, b and Supplementary Fig. S2a). Given that LDHB mainly catalyzes the conversion of lactate to pyruvate, we first investigated whether LDHB activity is important for lactate utilization. We performed rescue experiments, i.e., we determined the effect of lactate supplementation on the viability of cancer cells cultured in a medium containing only 2.5 mM of glucose (low-GLC) but no glutamine or pyruvate. Lactate supplementation for 4 days led to a relative increase in cell viability of A549-siCTRL cells but not after LDHB silencing (Fig. 2c). Furthermore, we also determined the uptake of ^13^C-labelled lactate by NMR as described before [35]. Silencing LDHB reduced lactate uptake from serum in A549 cells under low glucose conditions (Fig. 2d), suggesting that LDHB activity is required for lactate uptake. Since previous research indicated that lactate can be used as fuel for mitochondrial respiration and lactate is the primary fuel for the TCA cycle [19, 21], we speculated that lactate utilization mediated by LDHB contributes to OXPHOS. Indeed, acute silencing of LDHB significantly reduced basal, ATP-linked, and maximal OCR in a panel of NSCLC cell lines and the primary culture PF139 (Fig. 2e and Supplementary Fig. S2b, c). To better understand whether inhibition of LDHB after long-term adaptation reduces OXPHOS, we also generated shRNA-mediated LDHB silencing cells that exhibit dramatically reduced LDHB expression and activity (Fig. 2f, g). Not surprisingly, basal, ATP-linked, and maximal OCR were significantly decreased in A549 cells that stably expressed LDHB-specific shRNAs. (Fig. 2h). These results indicate that LDHB is essential for functional OXPHOS in the tested NSCLC cells.

It was shown before that inhibition of LDHA by a small molecule inhibitor decreases glycolysis and leads to a compensatory increase in OXPHOS [40]. Therefore, we speculated that silencing LDHB would increase ECAR. Surprisingly, silencing of LDHB decreased ECAR, i.e., a hallmark of glycolysis, in all tested cell lines, both after short- and long-term silencing (Supplementary Fig. S2d, g). In addition, glycolytic capacity and reserve were also reduced after LDHB silencing (Supplemental Fig. S2e), suggesting that the alteration in metabolism induced by LDHB inhibition is not identical to LDHA inhibition. In summary, silencing of LDHB in NSCLC cells reduced OCR and ECAR capacity, which was associated with a shift from an energetic to a more quiescent phenotype (Supplementary Fig. S2f).

### LDHB silencing reduces survival of NSCLC TICs and inhibits tumorigenesis and growth

**Fig. 3:**
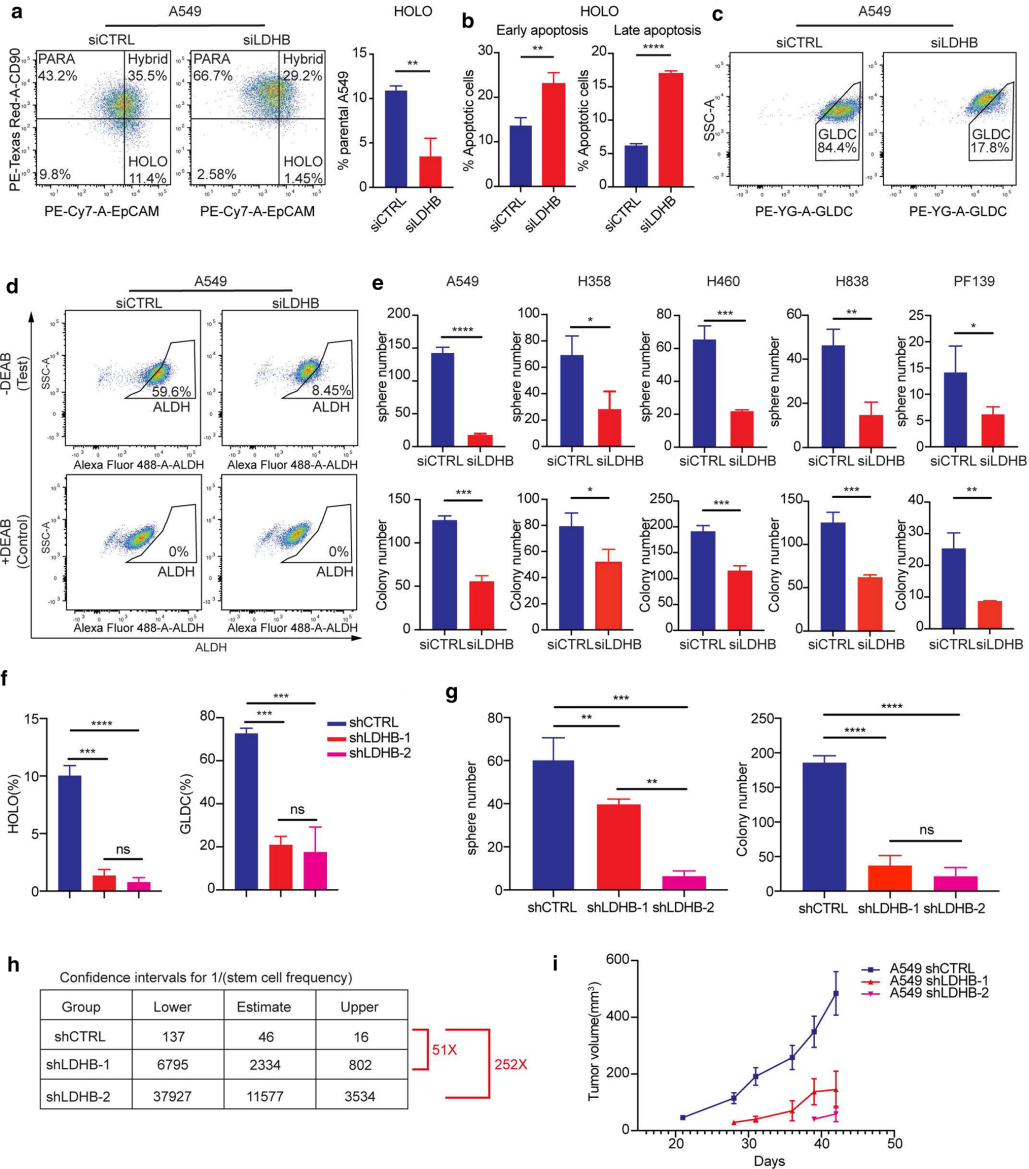
LDHB inhibition results in reduced survival of NSCLC TICs, tumor initiating capacity and growth. **a** Analysis of HOLO and PARA cells by flow cytometry using EpCAM and CD90 co-staining after 48 h of transfection with siCTRL or siLDHB. HOLO and PARA cells are represented as EpCAM + /CD90- and EpCAM-/CD90 + , respectively. Percentage of HOLO population in A549 cells was used to perform statistical analysis (*n* = 3). **P < 0.01, (two-tailed unpaired Student’s *t*-test). Error bars represent mean ± SD. **b** Analysis of apoptosis by flow cytometry using Annexin V and PI staining co-staining after 48 h of transfection with siCTRL or siLDHB. The early and late apoptotic population was shown by Annexin V + /PI − and Annexin V + /PI + , respectively (*n* = 3). **P < 0.01, ****P < 0.0001 (two-tailed unpaired Student’s *t*-test). Error bars represent mean ± SD. **c, d** Analysis of NSCLC TICs population by flow cytometer using staining with markers for TICs, e.g., GLDC and ALDH after 48 h of transfection with siCTRL or siLDHB. **e** 500 siCTRL or siLDHB cells were seeded in Nunclon Sphera 6-well plates and cultured with 3D CnT culture medium or 2D DMEM/F12 or RPMI medium after transfection with siCTRL or siLDHB. For the sphere formation assay, spheres were counted under the microscope, and for the colony formation assay, colonies were counted with Fiji after 7–21 days (*n* = 3). *P < 0.05, **P < 0.01, ***P < 0.001, ****P < 0.0001 (two-tailed unpaired Student’s *t*-test). Data are shown with mean ± SD. **f** Analysis of HOLO cells, and GLDC positive population by flow cytometry as same as described above (*n* = 3). *ns* no significant difference, ***P < 0.001, ****P < 0.0001 (Ordinary one-way ANOVA). The error bars are represented with mean ± SD. **g** Analysis of colony formation and sphere formation as described above (*n* = 3). *ns* no significant difference, **P < 0.01, ***P < 0.001, ****P < 0.0001 (Ordinary one-way ANOVA). Data are shown with mean ± SD. **h** Stem cell frequency was calculated based on extreme limited dilution analysis (details described in the methods section). **i** Growth curves were determined based on tumor volume at different timepoints

It has been shown that TICs are dependent on functional OXPHOS [18]. Indeed, the level of OXPHOS in A549 holoclonal cells was increased compared to paraclonal A549 cells (see Fig. 1a). Furthermore, silencing of LDHB significantly reduced OXPHOS, e.g., basal, maximal, and ATP-linked OCR in a panel of NSCLC cell lines and the primary culture PF139 (Fig. 2e and Supplementary Fig. S2b, c). Therefore, we speculated that inhibition of LDHB should result in decreased OXPHOS, thereby preferentially affecting TICs, e.g., the holoclonal subpopulation in the parental A549 cell line. Indeed, the fraction of holoclonal cells was decreased in the parental A549 cell line upon LDHB silencing (Fig. 3a). Interestingly, the fraction of paraclonal cells was also increased upon LDHB silencing (Fig. 3a and Supplementary Fig. S3a). To assess whether LDHB inhibition specifically affects TICs, we then performed LDHB silencing in purified cultures from holoclone and paraclone separately. We found that LDHB silencing reduced not only the proliferation of holoclone cells but also paraclonal cells (Supplementary Fig. S3b, left panel). However, the relative resistance to LDHB inhibition of the purified paraclonal culture was increased compared to the holoclonal culture (Supplementary Fig. S3b, right panel), indicating that the holoclonal status, which is associated with increased tumor initiation capacity, is highly sensitive to LDHB inhibition. Furthermore, silencing of LDHB not only reduced proliferation of holoclone cells but also increased early and late apoptosis (Fig. 3b and Supplementary Fig. S3c, left panel). To determine whether silencing LDHB leads to phenotypic plasticity in the remaining cells, we determined the expression of the epithelial and mesenchymal markers EpCAM and CD90, respectively. Indeed, silencing LDHB in A549 holoclonal cells significantly reduced the proportion of epithelial stem-like cells, e.g., EpCAM + CD90-, resulting in an increased proportion of cells with a meroclonal phenotype [33], e.g., EpCAM-CD90- (Supplementary Fig. S3c, middle and right panel). Finally, silencing of LDHB not only reduced the fraction of GLDC^+^ cells but also of ALDH^+^ cells in the A549, H358, and H460 cell lines (Fig. 3c, d and Fig. S3d, e).

In the NSCLC cell lines A549, H358, H460, H838, and the primary culture PF139, silencing of LDHB dramatically reduced colony and sphere formation, which serves as an in vitro surrogate assay to assess tumor initiation [35, 41] (Fig. 3e and Fig. S3f). Since LDHB is part of the *KRAS* amplicon [31], we speculated that the sensitivity to LDHB silencing might be limited to *KRAS* mutated cell lines. However, LDHB silencing also reduced sphere and colony formation of the NSCLC cell lines H520 and H1650, both of which harbor a wild-type form of *KRAS* (Supplementary Fig. S3f). In addition, in silico analysis of the lung adenocarcinoma patient’s samples available in the TCGA database revealed that LDHB expression correlated with the *TP53* mutational status (Fig. S3g). Thus, it is unlikely that sensitivity to LDHB inhibition depends solely on the mutational status of *KRAS*. Subsequently, the effect of long-term LDHB inhibition on NSCLC TICs was also investigated. The proportion of TIC-like cells, i.e., holoclonal cells as well as cells characterized by increased GLDC expression, remained lower after long-term adaptation to LDHB silencing compared with A549 shCTRL cells, despite the significant heterogeneity in EMT status among the A549 shLDHB clones examined (Fig. 3f and Supplementary Fig. S3h). Furthermore, the sphere and colony formation of A549-shLDHB cells continued to be suppressed (Fig. 3g and Supplementary Fig. S3i).

Strikingly, the tumor initiation capacity of the shLDHB-1 and shLDHB-2 clones was reduced by 51- and 252-fold compared to the A549 shCTRL clones (Fig. 3h and Supplementary Fig. S3j, right panel). In addition, after implantation of A549 shLDHB cells, the growth rate of the few initiated tumors remained markedly reduced compared with the control tumors (Fig. 3i). Similarly, in the few tumors initiated after implantation of A549 shLDHB cells, LDHB expression remained generally reduced compared with control tumors. However, although both LDHB and GLDC expression levels varied significantly between tumor regions (Supplementary Fig. S3j, left panel).

In addition, the proliferation of A549, H358, H460, and primary culture PF139 was also dramatically reduced after LDHB silencing (Supplementary Fig. S3k). Interestingly, in the cell line H838, which features the highest LDHB expression levels of the 135 NSCLC cell lines included in the cBioportal database, silencing of LDHB reduced sphere and colony formation, whereas proliferation was not affected (Fig. 3e and Supplementary Fig. S3f, k). These results suggest that different metabolic dependencies might characterize proliferation and tumorigenesis. Finally, silencing of LDHB did not significantly reduce proliferation, viability, and colony formation of the human non-tumorigenic lung epithelial cell line BEAS-2B (Supplementary Fig. S3l), which is consistent with a previous study showing that LDHB silencing reduces proliferation of several cancers but not normal cell lines [42]. Together, these results suggest an essential role for LDHB in NSCLC tumorigenesis and growth that may be cancer-specific.

### OXPHOS deficiency induced by LDHB inhibition is related to persistent mtDNA damage

**Fig. 4:**
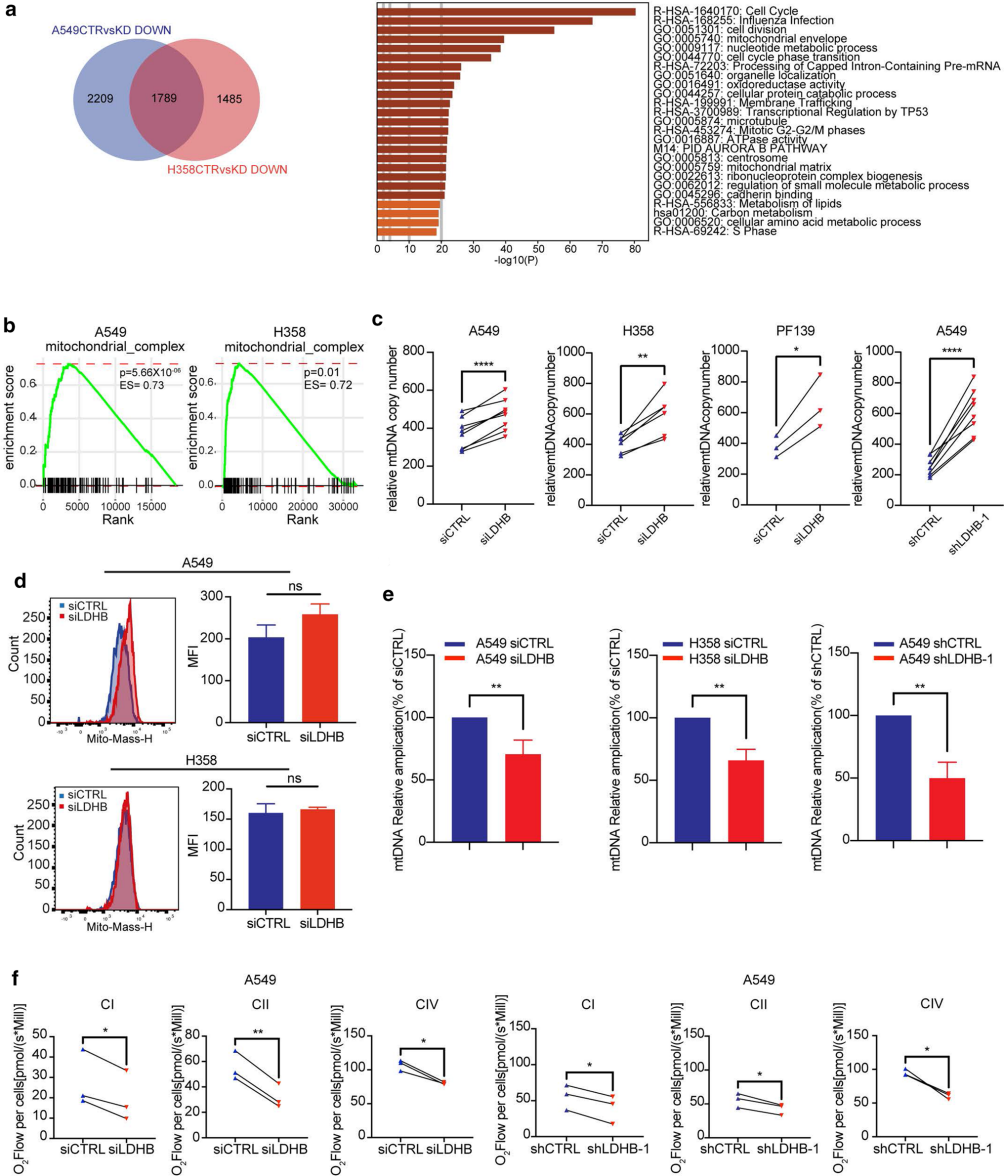
OXPHOS deficiency induced by LDHB inhibition is related to persistent mtDNA damage. **a** Venn diagram showing the number of genes down-regulated in A549 and H358 after LDHB silencing. Comprehensive pathway analysis showing top 25 down-regulated pathways or gene ontology terms in A549 and H358 after LDHB silencing. **b** Relative RNA expression-based enrichment of Mitochondrial Respiratory Chain Complexes in response to mitochondrial complex activity in siCTRL compared to siLDHB. The *y*-axis represents the enrichment value, and the *x*-axis represents the rank of differential expression for all genes, with group-specific genes indicated by vertical black lines. Rank positions to the left indicate increased expression in siCTRL. **c** mtDNA copy number were determined by qRT-PCR analysis on isolated DNA from siCTRL and siLDHB after 48 h of transfection or shCTRL and shLDHB cells (*n* = 3–8). *P < 0.05, **P < 0.01, ****P < 0.0001 (two-tailed paired Student’s *t*-test). The error bars represent mean ± SD. **d** Mitochondrial mass was analysis by flow cytometer using MitoTracker™ Deep Red FM after 48 h of transfection. Mean fluorescence intensity (MFI) was used for quantitative analysis (*n* = 3). *ns* no significant difference (two-tailed unpaired Student’s *t*-test). Error bars represent mean ± SD. **e** mtDNA damage were determined by qRT-PCR analysis on isolated DNA from siCTRL and siLDHB after 48 h of transfection or shCTRL and shLDHB cells (*n* = 3–8). **P < 0.01 (two-tailed paired Student’s *t*-test). The error bars represent mean ± SD. **f** Maximal ADP-stimulated respiration was quantified by OROBOROS to evaluate mitochondrial respiration complex I (CI), II (CII), IV (CIV) activity in A549 siCTRL and A549 siLDHB after 48 h of transfection or A549 shCTRL and shLDHB cells. The respiration rate is presented in pmol/(sec × million cells) (*n* = 3). *P < 0.05, **P < 0.01 (two-tailed paired Student’s *t*-test). The error bars represent mean ± SD

To investigate the molecular mechanisms underlying the pleiotropic cellular changes induced by LDHB silencing, we performed a comprehensive whole transcriptome expression analysis of the *KRAS*-mutated A549 and H358 cell lines. Silencing LDHB significantly reduced the expression of 1789 genes in both A549 and the H358 cells (Fig. 4a, left panel), which enriched in the pathways associated with the mitochondrial morphology, e.g., mitochondrial envelope and mitochondrial matrix (Fig. 4a, right panel), which was in agreement with the co-localization of LDHB with mitochondria in A549 and H460 cells (Supplementary Fig. S4a) and a previous report in HeLa cells [27]. Gene set enrichment analysis (GSEA) based on Hallmark gene sets also shows that the oxidative phosphorylation pathway is significantly enriched in scramble control cells compared with LDHB-silencing cells in A549 and H358 cell lines (Supplementary Fig. S4b), consistent with our results showing that LDHB silencing inhibits OXPHOS. These analyses indicate that LDHB is important for mitochondrial function.

The electron transport chain (ETC) of the mitochondrial oxidative phosphorylation system consists of ~ 80 polypeptides, most of which are nuclear-encoded, except for the 13 core subunits encoded by mitochondrial DNA, which additionally encodes two ribosomal RNAs and 22 transfer RNAs necessary for translation of the 13 proteins [43]. Our GSEA based on the HUGO Gene Nomenclature Committee (HGNC) mitochondrial respiratory chain complex gene group revealed that genes related to mitochondrial respiratory chain complexes were significantly enriched in scramble control cells compared with LDHB-silencing cells in A549 and H358 cell lines (Fig. 4b). This analysis suggests that LDHB silencing reduces the expression of both nuclear and mitochondrial DNA-encoded mitochondrial genes, consistent with the reduced OXPHOS of the cancer cell lines tested (see Fig. 2). Intriguingly, the expression of CMPK2, which provides nucleotides for mtDNA repair and replication [44], is among the genes whose expression increased most upon LDHB silencing in both the A549 and the H358 cell lines (expression increased more than six fold, see supplementary Excel file Gene Expression). We, therefore, hypothesized that OXPHOS deficiency induced by silencing LDHB was associated with impairment of mitochondrial DNA integrity. To our surprise, mitochondrial DNA copy numbers were consistently increased both after short-term silencing in H358 and the primary NSCLC culture PF139 and long-term silencing in A549 cells (Fig. 4c). Mitochondrial mass was also not reduced after LDHB silencing (Fig. 4d). However, short-term silencing of LDHB decreased the relative amplification of non-damaged mtDNA in the A549 and H358 cell lines, which was also observed after long-term silencing in A549 cells (Fig. 4e). This suggests that silencing of LDHB induces mtDNA damage that cannot be compensated by an increase in mitochondrial DNA copy numbers, which is in agreement with the reduced expression of genes encoded by mitochondrial DNA (Fig. 4b). However, protein levels of the electron transport chain complexes were not significantly reduced upon LDHB silencing, at least not 48 h after the induction of LDHB silencing (Fig. S4d). Nevertheless, the activity of individual mitochondrial ETC complexes I and IV, encoded by mitochondrial DNA, and complex II, encoded by nuclear DNA, was reduced after short-term silencing of LDHB and remained reduced regardless of substrate availability during long-term adaptation (Fig. 4f and Supplementary Fig. S4c), indicating that the OXPHOS capacity per se is reduced independent of the supply of the TCA-derived electron carrier fueling OXPHOS, e.g., NADH. In this context, it was shown that both NAD^+^ and NADH levels were dramatically increased in OXPHOS-deficient Rho 0 cells upon pyruvate starvation [45]. Indeed, both NAD + and NADH levels were significantly increased after long-term LDHB silencing (Supplementary Fig. S4e).

Thus, these findings further support our hypothesis that persistent mitochondrial DNA damage induced by LDHB silencing might be the underlying molecular feature responsible for the inability of a cancer cell to adapt their mitochondrial-encoded OXPHOS and consequently their mitochondria-dependent metabolism, thereby abolishing the tumor initiation capacity.

### LDHB inhibition downregulates mitochondrial-dependent pyrimidine and purine synthesis pathways

**Fig. 5:**
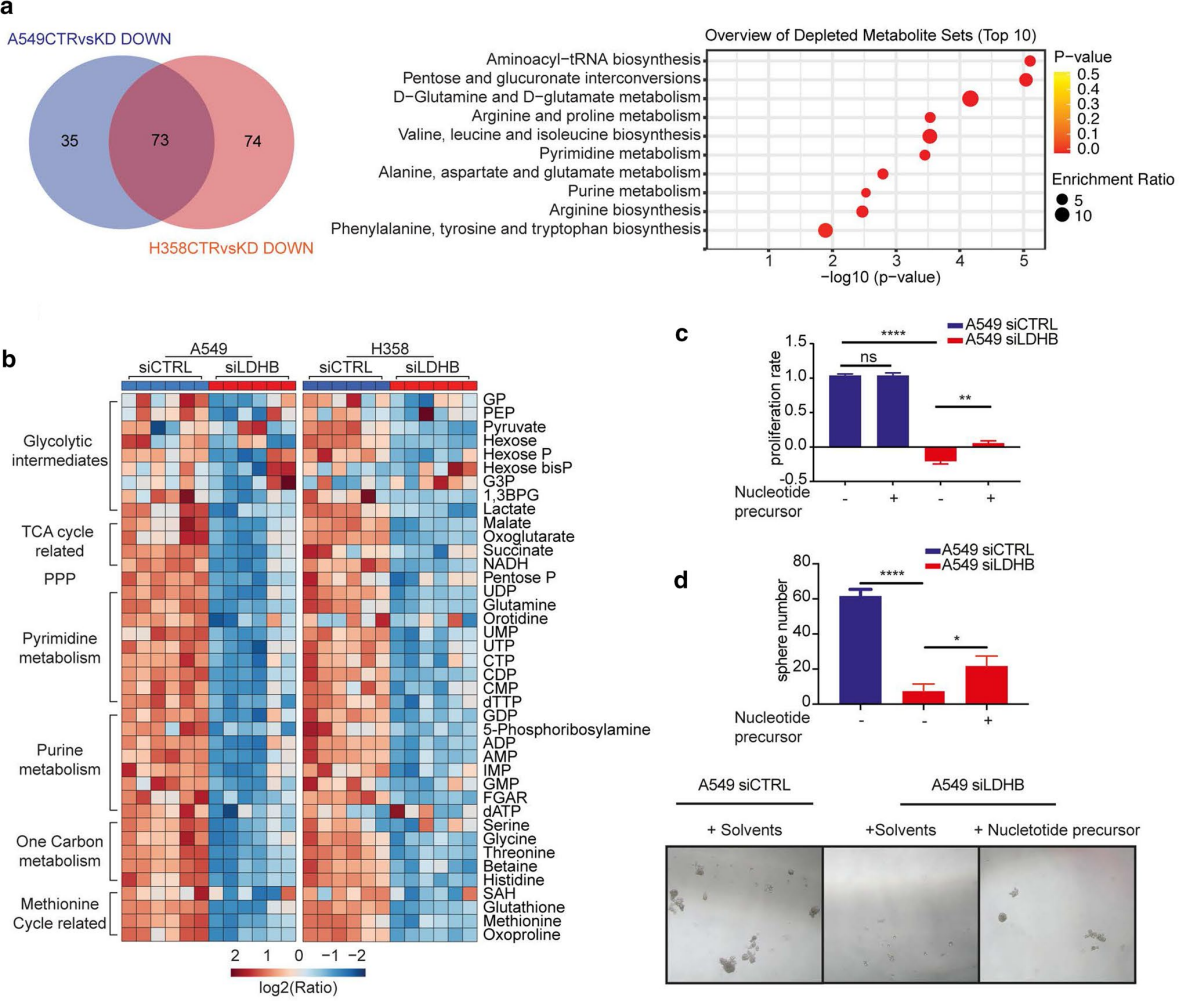
LDHB inhibition downregulates mitochondrial-dependent pyrimidine and purine synthesis pathways. **a, b** Venn diagram showing the number of metabolites reduced in A549 and H358 after LDHB silencing. Balloon plot showing metabolite enrichment analysis (MSEA) of reduced metabolites in A549 and H358 after LDHB silencing. The top 10 dysregulated metabolic pathways are shown. The size and color of the balloon indicate the enrichment ratio and *P*-value, respectively. Heatmap showing the metabolomic comparison of siCTRL and siLDHB cells. Six replicates (including three biological replicates with two technical replicates) are shown as separate columns for each cell type. log2 of the ratio between the metabolite intensity of each sample to the average intensity of all samples. **c, d** For proliferation and sphere formation ability, rescue experiments were performed with the addition of the following nucleotide precursors: 100 µM hypoxanthine, 100 µM adenine, 400 µM uridine. The number of cells and spheres was counted after 5 and 7 days with nucleotide precursor rescue (*n* = 3). *ns* no significant difference, *P < 0.05, **P < 0.01, ****P < 0.0001 (Ordinary two-way ANOVA). The error bars represent mean ± SD

It is well established that functional mitochondria are essential for nucleotide metabolism, which is important for tumorigenesis [18, 46–49]. Therefore, we performed metabolomics analysis through an untargeted liquid chromatography–mass spectrometry (LC–MS) to corroborate our gene expression analysis data and provide additional unbiased and comprehensive insights into how LDHB silencing affects cancer cells and whether LDHB links mitochondrial metabolism to nucleotide metabolism. Of the detected metabolites, silencing of LDHB increased the levels of 188, 6 remained unchanged, whereas the levels of 196 metabolites increased. Metabolite set enrichment analysis (MSEA) revealed that "Biosynthesis of unsaturated fatty acids” was the only KEGG pathway significantly enriched in the group of metabolites elevated in both A549 and H358 cells (Supplementary Fig. S5a). In agreement with the data of our gene expression analysis, levels of metabolites and genes related to the mitochondrial-dependent metabolism were generally reduced after silencing of LDHB, e.g., TCA cycle and amino acid (Fig. 5b and Supplementary Fig. S5b-d), supporting our conclusion that silencing of LDHB targets mitochondria-related metabolism.

In both A549 and H358 cell lines, silencing of LDHB significantly reduced 73 metabolites that are significantly enriched in the nucleotide metabolic pathway, such as pyrimidine and purine metabolism (Fig. 5a), indicating that silencing LDHB reduces nucleotide metabolism. In detail, the level of nucleotides associated with pyrimidine metabolism, such as UDP, UMP, UTP, CTP, CDP, dTTP, reduced dramatically, and also glutamine level which is a substrate for pyrimidine synthesis (Fig. 5b and Supplementary Fig. S5d). Meanwhile, the level of nucleotides and substrates associated with purine metabolism, such as IMP, AMP, ADP, GDP, 5-phosphoribosylamine, and FGAR, also reduced dramatically upon LDHB silencing (Fig. 5b and Supplementary Fig. S5d). In addition, metabolites associated with mitochondria-dependent one-carbon and methionine cycle metabolism, which are important for nucleotide synthesis, are also reduced (Fig. 5b). Our in silico analysis of data previously published by Quin et al. [50] showed that reduction of LDHB expression by treatment with R-2HG or with siRNA, as performed in our current study, resulted in nearly identical depletion of metabolites (Supplementary Fig. S5e, left panel). Indeed, out of the 45 metabolites depleted after LDHB silencing, 30 were also depleted after R-2HG treatment. Those metabolites were most significantly related to pyrimidine and purine metabolism, e.g., nucleotide synthesis (Supplementary Fig. S5e, right panel). This analysis further validates our conclusion that LDHB inhibition in NSCLC cells results in reduced mitochondria-dependent biogenesis, especially pyrimidine and purine metabolism.

To verify whether reduced nucleotide is responsible for inhibited tumor initiating capacity and proliferation, we subsequently performed a rescue experiment by supplementation with nucleotide precursors, e.g., hypoxanthine, uridine, adenine. The addition of exogenous nucleotide precursors did not increase the maximal proliferation of siCTRL-transfected A549 cells but did significantly increase the reduced proliferation rate after LDHB silencing. (Fig. 5c and Supplementary Fig. S5f). Indeed, nucleotide supplementation also partially rescued sphere formation upon LDHB silencing in A549 cells (Fig. 5d). In summary, the results show that LDHB silencing in NSCLC cells significantly impairs nucleotide synthesis, which is required for proficient proliferation and sphere formation of NSCLC cells.

### Lactate supplementation increases survival of patient-derived tumor tissue ex vivo

**Fig. 6:**
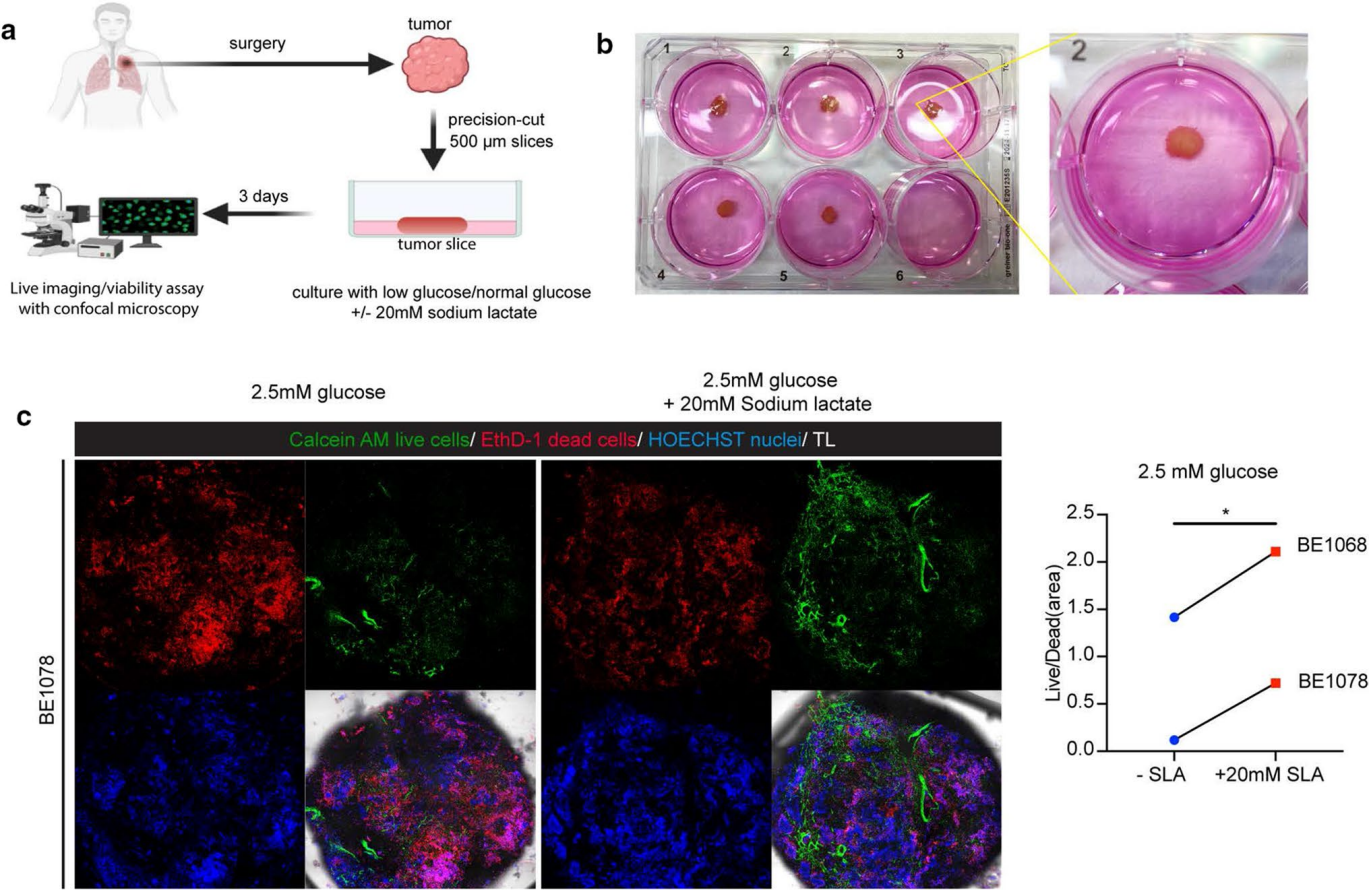
Lactate supplementation increases survival of patient-derived tumor tissue ex vivo. **a, b** Schematic representation of an experiment, where precision cut tumor slices (PCTS) was cultured in DMEM medium with or without 20 mM sodium lactate. **c** Analysis of cell viability of PCTS after addition of 20 mM sodium lactate in DMEM medium containing 2.5 mM glucose after 3 days by Calcein AM staining (green) for live cells, EthD-1 staining (red) for dead cells and HOECHST (blue) for DNA. The images were taken with a Zeiss LSM 880 confocal microscope and analyzed by Fiji. The Live/Dead ratio is presented (*n* = 2). *P < 0.05 (two-tailed paired Student’s *t*-test)

Our in silico analysis revealed that LDHB expression was higher in tumor tissue than in normal tissue in both lung adenocarcinoma (LUAD) and squamous cell carcinoma (LUSC) and increased LDHB expression in LUAD correlated with poor patient survival (Supplementary Fig. S6a, b). Thus, we extended our analysis of lactate-related metabolism to patient-derived tumor tissue cultured ex vivo (Fig. 6a, b). LDHB mainly converts lactate to pyruvate, so the function of LDHB is inextricably linked to lactate metabolism. Indeed, lactate supplementation partially rescued the decrease in proliferation and sphere formation induced by LDHB silencing (Fig. 5c, d). Therefore, we supplemented cell culture medium containing 5 mM or 10 mM glucose with 20 mM sodium lactate, the physiological lactate concentration of healthy and inflamed tissues including the tumor microenvironment [51]. We found that lactate supplementation significantly increased lung tumor cell survival (Fig. 6c and Supplementary Fig. S6c), corroborating that lactate metabolism is critical for human NSCLC cell survival.

### Depletion of LDHB results in decreased tumorigenesis and delayed tumor growth in a genetically engineered mouse model of NSCLC

**Fig. 7:**
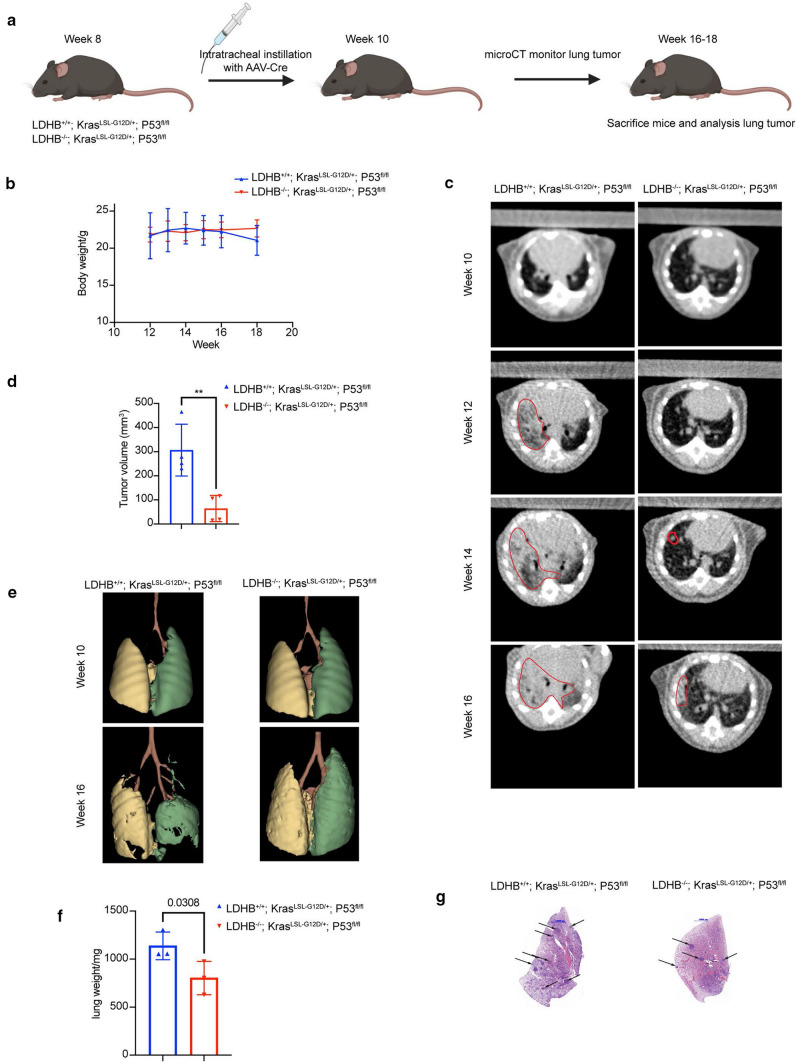
Depletion of LDHB results in decreased tumorigenesis and delayed tumor growth in a genetically engineered mouse model of NSCLC. **a** Schematic representation of the lung tumor induction experiment in LDHB knockout and wild-type mice. **b** Bodyweight was determined from LDHB^+/+^; K-ras^LSL−G12D/+^; p53^fl/fl^ and LDHB^−/−^; K-ras^LSL−G12D/+^; p53^fl/fl^ mice at different timepoints (*n* = 3). The error bars represent mean ± SD. **c** Representative axial CT scans from LDHB^+/+^; K-ras^LSL−G12D/+^; p53^fl/fl^ and LDHB^−/−^; K-ras^LSL−G12D/+^; p53^fl/fl^ mice after intratracheal instillation with AAV-Cre virus at different timepoints. **d** Tumor volume of LDHB^+/+^; K-ras^LSL−G12D/+^; p53^fl/fl^ and LDHB^−/−^; K-ras^LSL−G12D/+^; p53^fl/fl^ mice was quantified at 16 weeks of age by 3D Slicer software (*n* = 4). **P < 0.01 (two-tailed unpaired Student’s *t*-test). The error bars represent mean ± SD. **e** 3D view of lungs from LDHB^+/+^; K-ras^LSL−G12D/+^; p53^fl/fl^ and LDHB^−/−^; K-ras^LSL−G12D/+^; p53^fl/fl^ mice at 16 weeks of age (8 weeks after intratracheal instillation with AAV-Cre). **f** Lungs from LDHB^+/+^; K-ras^LSL−G12D/+^; p53^fl/fl^ and LDHB^−/−^; K-ras^LSL−G12D/+^; p53^fl/fl^ mice were harvested and weighed after 8 weeks of intratracheal instillation with AAV-Cre virus (*n* = 3). A two-tailed unpaired Student’s *t*-test was used for statistical analysis. The error bars represent mean ± SD. **g** Representative HE images are from the mice sacrificed above

In contrast to a conventional mouse xenograft tumor model, GEMMs enable the initiation of orthotopic tumors that best mimic the pathological conditions of human tumors in terms of immune compatibility and tumor microenvironment [52]. Therefore, to better understand the role of LDHB in NSCLC tumorigenesis, we developed a preclinical GEMM of NSCLC in which lung tumors can be induced by induction of *KRAS* mutation and *TP53* deletion in addition to LDHB depletion or wild-type status, e.g., LDHB^+/+^; K-ras^LSL−G12D/+^; p53^fl/fl^ and LDHB^−/−^; K-ras^LSL−G12D/+^; p53^fl/fl^ mice, its genotype was verified by Southern blot (Fig. 7a, Supplementary Fig. S7a). A complete hereditary deficiency of LDHB expression was identified in two patients [53]. Furthermore, the homozygous deletion of LDHB in the mouse model results only in a minor phenotype, e.g., increased lean body mass, decreased total body fat/circulating insulin, but no immunodeficiency was observed (www.mousephenotype.org). Therefore, we speculated that LDHB deletion could be tolerated without having a significant effect on the animal health status. Indeed, we did not observe a difference in body weight between LDHB^+/+^; K-ras^LSL−G12D/+^; p53^fl/fl^ and LDHB^−/−^; K-ras^LSL−G12D/+^; p53^fl/fl^ animals (Fig. 7b). Our in silico analysis based on a GEO data set also revealed that LDHB expression was increased in oncogene-induced lung dysplasia compared to normal lung tissue (Supplementary Fig. S7b). Indeed, tumor-initiation was detectable in LDHB^+/+^; K-ras^LSL−G12D/+^; p53^fl/fl^ mice at 12 weeks of age, 4 weeks after intratracheal instillation, whereas no tumor initiation was detectable in control animals (Fig. 7c and Supplementary Fig. S7c). Two weeks later, a reduced number of tumors was also detectable in LDHB^−/−^; K-ras^LSL−G12D/+^; p53^fl/fl^ animals (Fig. 7c and Supplementary Fig. S7c). At week 16, the tumor volume was significantly reduced in LDHB^−/−^; K-ras^LSL−G12D/+^; p53^fl/fl^ animals compared to control animals (Fig. 7d). These results suggest that depletion of LDHB inhibits tumorigenesis and delays tumor growth. We also observed lower disease incidence in the lungs of LDHB^−/−^; K-ras^LSL−G12D/+^; p53^fl/fl^ mice with a 3D reconstruction of micro-CT images (Fig. 7e). Moreover, at the end of the experiment (week 17), the average weight of lungs from LDHB^−/−^; K-ras^LSL−G12D/+^; p53^fl/fl^ animals was significantly lower, and fewer tumor nodules were detectable compared to those from LDHB^+/+^; K-ras^LSL−G12D/+^; p53^fl/fl^ (Fig. 7f, g), which further confirmed our conclusion that LDHB is important for NSCLC tumorigenesis.

## Discussion

Guided by the metabolic symbiosis model (reviewed in Ref. [22], we hypothesized that, in the A549 cell line, the tumor-initiating holoclonal subpopulation is dependent on LDHB to metabolize the lactate produced by the more mesenchymal paraclonal subpopulation [33]. Indeed, LDHB silencing not only diminished the fraction of holoclonal A549 cells but also decreased the fraction of GLDC^+^ cells (Fig. 3c). Intriguingly, lactate preferentially rescued the survival of the holoclonal A549 subpopulation and increased the fraction of the GLDC^+^ subpopulation (Fig. 1c, d and Supplementary Fig. S1e). In the context of maintaining a stem-like phenotype, it was shown that retinoid (vitamin A) is oxidized to Retinal, which serves as a substrate for ALDH1 to synthesize retinoic acid, e.g., a metabolite essential for differentiation during early embryonic development and the maintenance of cancer stem cell subpopulations in tumors (reviewed in Ref. [54]. In agreement with the dramatically reduced aldehyde activity in all three-tested cell lines (Fig. 3d and Supplementary Fig. S3e), retinoid was one of the most abundantly accumulated metabolites upon LDHB silencing (supplementary Excel file Metabolomics Data). Furthermore, expression of the most abundantly expressed aldehyde dehydrogenase isoforms, e.g., ALDH1A1 and ALDH3A1, which are 1.6- and 7.1-fold overexpressed in the stem-like holoclonal compared to the more mesenchymal paraclonal A549 cells [33], was also significantly reduced upon LDHB silencing (supplementary Excel file Gene Expression). Thus, in agreement with reducing the GLDC^+^ and ALDH^+^ subpopulations, LDHB silencing reduced the synthesis of pyrimidine and retinoic acid, both of which are critically associated with maintaining a stem-like state linked with increased tumor initiation capacity. However, additional studies will be necessary to elucidate on the molecular level how the modulation of LDHB expression affects the complex interplay between lactate metabolism and stemness pathways that are associated with an increased tumor initiation capacity.

It has been shown that silencing of LDHA decreases glycolysis and leads to a reactivation of mitochondrial function in breast, lung, liver, lymphoma, and pancreatic cancers (reviewed in Ref. [55]. Thus, we speculated that silencing LDHB should result in lactate accumulation and blunting of mitochondrial metabolism. Indeed, LDHB silencing decreased OCR (Fig. 2e). Surprisingly, intracellular lactate levels were also significantly decreased upon LDHB silencing (supplementary Excel file Metabolomics Data), as was lactate-dependent ECAR (Supplementary Fig. 2d). Lactate is the final product of glycolysis. Thus our results suggested that LDHB silencing inhibits glycolysis. Indeed, the levels of glycolytic intermediates were significantly depleted upon LDHB silencing, as were the levels of metabolites of the PPP, which is fueled by glycolytic intermediates (Fig. 5b). In agreement, our Seahorse analysis indicated that glycolysis is significantly inhibited upon LDHB silencing in all tested cell lines (Supplementary Fig. S2d). In summary, short-term silencing of LDHB, in contrast to inhibition of LDHA, inhibited both mitochondrial metabolism and glycolysis. Interestingly, long-term silencing of LDHB also decreased LDHA protein expression (Fig. 2f), suggesting that feedback mechanisms may regulate the metabolic rewiring that occurs during long-term adaptation.

Interestingly, mitochondrial DNA integrity was not restored after LDHB silencing (Fig. 4e), so LDHB silencing resulted in a phenotype similar to that observed in Rho 0 cells. In detail, long-term treatment with a low dose of the DNA damage-inducing agent Ethidium Bromide results in complete depletion of mitochondrial DNA, the definition of Rho 0 cells [56]. Consequently, Rho 0 cells rely on medium supplementation with uridine to compensate for the loss of the ETC complex III, which is encoded by mitochondrial DNA and is essential for the function of DHODH, e.g., pyrimidine de novo synthesis. Intriguingly, although Rho 0 cancer cells retained their proliferation ability, they completely lost their tumor initiation capacity [57], thus resembling cancer cells adapted to long-term LDHB silencing, e.g., A549 shLDHB clones (data not shown). Strikingly, the generation of tumors in syngeneic mice by Rho 0 cancer cells is linked to the acquisition of the host mtDNA from stromal cells and subsequent restoration of the OXPHOS activity [58]. Indeed, we also observed that tumor initiation was dramatically reduced but not completely abrogated, both in xenografts of A549 shLDHB clones (Fig. 3h) and also in LDHB knockout animals (Fig. 6c). Thus, it will be interesting to elucidate how cellular metabolism can be restored upon LDHB silencing, e.g., either by the acquisition of mitochondria or by rewiring the cellular metabolism.

At the metabolic level, proliferation and sphere formation were only partially rescued by nucleotide supplementation after LDHB silencing (Fig. 5), suggesting that the effect of silencing LDHB is not limited to nucleotide synthesis. Indeed, our metabolomic analysis showed that short-term silencing of LDHB resulted in a reduction in TCA intermediates, which was associated not only with reduced mitochondria-dependent biogenesis, e.g., of nucleotides and amino acids, but also with reduced production of energy carriers. This suggests that LDHB-mediated metabolism is not only critical for nucleotide synthesis, but also represents a critical bottleneck for the production of a variety of mitochondria-related metabolites in NSCLC cells.

Regarding the differences in metabolic states, a shortcoming of our study is that we performed neither the gene expression analysis nor the metabolomics at the single-cell level. Indeed, single-cell RNA-sequencing of cancer cells revealed significant differences in LDHB expression between and within cancer, stroma, and immune subpopulations of primary breast cancer tumors [59] (see also https://www.ebi.ac.uk/gxa/sc/experiments/E-GEOD-75688/results?geneId=ENSG00000111716). Our microscopic analysis revealed that LDHB expression in the xenografts was highly heterogeneous (Supplementary Fig. S3j), confirming that lactate-mediated metabolic symbiosis might occur not only between cancer cells but also between all cell types within a tumor. Nevertheless, lactate supplementation generally increased survival in patient-derived ex vivo tumor tissues (Fig. 6c and Supplementary Fig. 6c), consistent with the finding that lactate is the primary carbon source for the TCA cycle in vivo and provides substrate and electrons for oxidative phosphorylation [20, 21]. It will be interesting to dissect how the underlying genetic background and the various gradients, e.g., pH, hypoxia, metabolites, affect lactate-mediated metabolic symbiosis between single cells and the cellular subpopulations in the complex tumor microenvironment.

In the context of persistent mtDNA damage, mitochondrial DNA was recently shown to serve as a cellular genotoxic stress sentinel [44]. Intriguingly, failure to restore mtDNA integrity may be an active process leading to the release of damaged mtDNA into the cytoplasm, thereby serving as a second messenger signaling cellular stress [44]. Specifically, once released into the cytoplasm, mtDNA activates not only the cyclic GMP–AMP synthase (cGAS)–STING (stimulator of interferon genes) dependent innate immune signaling pathway but also activates Toll-like receptor 9, inflammasomes (most notably Nlrp3), and other nucleic acid sensors in the cytoplasm [44]. Consistently, we found that most of the signaling pathways whose gene expression was increased after LDHB silencing were related to immune activation (Supplementary Fig. S4b). However, the mechanisms underlying the release of mtDNA into the cytoplasm have not yet been defined, nor have the numerous up- and downstream effectors that orchestrate the sentinel function of the mtDNA release. In addition, LDHB silencing affects lactate metabolism and pH regulation, both of which are intrinsically linked to the regulation of the immune response [60]. Thus, our study suggests that by triggering the sentinel function of mtDNA upon its depletion, LDHB may serve as an integral component of an evolutionarily conserved and actively maintained pathway to trigger immune activation in the presence of aberrant lactate metabolism.

In summary, our results lead us to propose a working model: consistent with the “reverse Warburg effect” model, a mesenchymal subpopulation of NSCLC cells exhibits increased glucose uptake, which is converted to lactate via LDHA-dependent glycolysis. The secreted lactate fuels NSCLC TICs featuring cancer stem cell markers, e.g., increased GLDC expression, OXPHOS, and ALDH activity. NSCLC TICs convert lactate in an LDHB-dependent manner to pyruvate, which subsequently drives mitochondria-dependent metabolism, including nucleotide synthesis required for nuclear and mtDNA replication and maintenance, as well as NAD^+^/NADH synthesis. Mitochondrial DNA integrity cannot be restored, which is associated with a continuously reduced mitochondrial complex activity and OXPHOS.

Interestingly, the activity of individual mitochondrial complexes encoded by mitochondrial DNA remained reduced regardless of substrate availability during long-term adaptation (Fig. 4f and Supplementary Fig. S4c), suggesting that it is indeed the OXPHOS capacity per se that is reduced, rather than the supply of OXPHOS substrates, i.e., the energetic products of the TCA cycle. Decreased OXPHOS activity further decreases pyrimidine synthesis, reducing stemness and thus sphere and tumor initiation ability. Furthermore, our results revealed that LDHB silencing triggers an innate immune response. We speculate that LDHB silencing leads to deregulation of mtDNA replication, which may elicit the release of mtDNA into the cytosol, thereby triggering an STING-dependent innate immune response [61], as recently shown in response to cellular pyrimidine imbalance [62]. However, further experiments are required to elucidate the details of the molecular mechanisms underlying the pleiotropic cellular changes induced by LDHB silencing in NSCLC cells.

In summary, our work contributes to the growing body of evidence suggesting that lactate metabolism is an important determinant of cancer stem cell maintenance and that nucleotide metabolism at the molecular level is inextricably linked to an increased tumor initiating capacity and tumorigenesis. Surprisingly, our study also revealed a link between LDHB-mediated lactate metabolism and the persistent accumulation of DNA damage of mitochondrial DNA, which is not only associated with a reduced capacity for tumorigenesis, mimicking Rho 0 cells but also with the activation of innate immune response. Thus, our study may provide the basis for the development of future therapies based on targeting LDHB in combination with agents that affect cellular plasticity, induction of DNA damage, or even immunotherapy.

## Methods

### Cell culture

All cell lines were obtained from American Type Culture Collection (Manassas, VA, USA), except patient-derived primary LUAD cells PF139 were established as recently reported [63]. Cells were maintained in the RPMI medium (Cat. #8758; Sigma-Aldrich) or DMEM/F12(Cat. #21331020; Life Technologies) supplemented with 2 mM L-glutamine (Cat. #25030024, Life Technologies); 10% fetal bovine serum (Cat. #10270-106; Life Technologies) and 1% penicillin/streptomycin solution (Cat. #P0781, Sigma-Aldrich). The cells were authenticated by DNA fingerprinting and confirmed free from mycoplasma contamination (Microsynth, Bern, Switzerland) at 37 °C in a humidified 5% CO_2_ incubator. Cell numbers were determined using a hemocytometer and 0.1% trypan blue for dead cell exclusion.

### Oxygen consumption rate (OCR) and extracellular acidification rate (ECAR)

Oxygen consumption rate and extracellular acidification rate were measured using the XFe96 Extracellular Flux Analyzer (Agilent Technologies). 18,000 PARA, A549, H838, and 20,000 HOLO, H358, H460, PF139 cells were seeded overnight in Seahorse XF96 V3 PS cell culture microplates (Cat. #101085-004; Agilent Technologies) and reached 80–90% confluence on the day of the experiment. The XF sensor cartridges were hydrated overnight with sterile ddH2O in a CO_2_-free incubator at 37 °C. For Mito Stress Test, cells were washed twice and changed into Seahorse XF DMEM or RPMI medium (Cat. #103680-100 and 103681-100, Agilent Technologies) containing 10 mM glucose, 0.5 mM pyruvate, 2 mM glutamine adjusted to pH 7.4. Then the cells were incubated in a CO_2_-free incubator for 1 h. Then, 1 μM oligomycin, 1.0 μM and 1.5 μM FCCP, a mixture of 1 μM rotenone and 1 μM antimycin A were added successively. For the Glycolysis Stress Test, cells were washed twice and changed into Seahorse XF DMEM, or RPMI medium containing 2 mM glutamine and adjusted to pH 7.4. The cells were then incubated in a CO2-free incubator for 1 h. Then 10 mM glucose, 1 μM oligomycin, 50 mM 2-DG were added successively. All chemicals are listed in Supplementary Table S2. All Seahorse measurements were conducted as at least three biological experiments, each with 7–26 technical replicates. The data were analyzed with Seahorse Wave (Agilent Technologies). All raw data is normalized to 50 ng DNA, which is quantified by CyQUANT™ Cell Proliferation Assay kit (Cat. # C7026; Thermo Fisher Scientific) according to the manufacturer’s protocol. Respiration parameters were determined as follows: Basal respiration as basal OCR was calculated as the mean of the 4 timepoints; ATP-linked respiration by subtracting proton leak from basal OCR; spare respiration as the difference between maximal respiration and basal respiration; maximal OCR calculated as the difference of antimycin plus rotenone rate from FCCP rate. Basal ECAR as glycolysis; maximal glycolysis and glycolytic capacity achieved by the additional oligomycin injection; glycolytic reserve as the difference between maximal and basal ECAR. Data from Seahorse experiments representing OCR or ECAR measurements over time are presented as arithmetic mean ± SEM of representative individual biological experiments.

### 2D colony formation assay and 3D sphere formation assay

For the 2D colony formation assay, 200–500 cells were cultivated in 6-well plates for 7–14 days. Colonies were stained with crystal violet (0.5% dissolved in 25% methanol). Images of the plates were then acquired using a Kaiser eVision executive High Frequency Illuminated Copy Stand to avoid shadows. To determine the number of colonies per well, the images were then analyzed using Fiji software (Fiji, RRID:SCR_002285). In detail: First, ROIs were set according to the boundary of the wells in the plate and the outer area was removed. In a second step, the original images were converted to 8-bit grayscale and then brightness/contrast were manually adjusted to identify the edges of the colonies. In a third step, colonies were separated from the background by manually setting a threshold depending on the individual background of the images. Then, the binary process feature was used to fill holes within each colony. In the next step, the watershed algorithm was applied to subdivide contiguous clones. Finally, the number of colonies was determined by applying the Analyze Particles feature, using the following size and circularity parameters: size: 50—infinity, circularity: 0.2–1.

For the 3D sphere formation assay, 500 cells were cultured in 2 mL CnT-Prime Airway Epithelial Proliferation Medium (Cat. #CnT-PR-A; CELLNTEC) supplemented with 0.5% methylcellulose (Cat. #M0262; Sigma-Aldrich); 10 ng/mL Human IGF-II (Cat. #100-12; PeproTech); 10 ng/mL Human Heregulinβ-1 (Cat. #AF-100-03; PeproTech); 1 μM DMH-1 (Cat. #73632; STEMCELL Technologies); 1 μM A-83-01 (Cat. #72022; STEMCELL Technologies); 1% penicillin/streptomycin solution (Cat. #P0781, Sigma-Aldrich); in Nunclon Sphera 6-Well Plate (Cat. #174932; Thermo Fisher Scientific). After 2–3 weeks, spheres were counted under the microscope. To be counted as a sphere, the individual spheres must have a size of more than 20 individual cells, confirming the growth of the sphere as opposed to background aggregates of, non-dividing, individual cells. In addition, the sphere must have a bright appearance, indicating that the cells of the sphere are viable. Images were taken at 40X magnification.

### Nucleotide rescue experiment

For the proliferation rescue experiment, cells were plated in triplicate into 6-well plates with an initial seeding density of 0.1 × 10^6^ cells per well for overnight equilibration. Subsequently, cells were changed to a medium with 100 μM hypoxanthine (Cat. # H9377; Sigma-Aldrich), 100 μM adenine (Cat. #A8626, Sigma-Aldrich), 400 μM uridine (Cat. #U6391; Sigma-Aldrich), or solvents (NaOH and HCl). According to the established methods or solvents (NaOH and HCl). According to the established procedure, cell number was quantified after 5 days using a hemacytometer (Cat. #Z359629; Merck) [64]. The proliferation rate was calculated using the following formula:

$$\begin{gathered} {\text{Proliferation rate }}\left( {\text{doublings per day}} \right) \hfill \\ \quad = {\text{ log}}_{{2}} \left( {{\text{final cell count}}\left( {\text{day 5}} \right)/{\text{initial cell count}}\left( {\text{day 1}} \right)} \right)/{4 }\left( {{\text{days}}} \right) \hfill \\ \end{gathered}$$

For the sphere formation rescue experiment, 500 cells were cultured in the CnT-Prime medium, as described above, with the indicated concentration of hypoxanthine, adenine, uridine, or solvents. After 2–3 weeks, the spheres were counted as described above.

### Flow cytometry

Cells were harvested as indicated above. Cells were stained with antibodies against surface markers for 30 min on the ice for the extracellular staining, protected from light. For the intracellular staining, cells were fixed with IC fixation buffer (Cat. #00-8222-49; Thermo Fisher Scientific) for 15 min and permeabilized with 0.1% Triton X100 (Cat. #X100; Sigma-Aldrich) for 10 min at room temperature. Then, cells were incubated in 200 μL PBS containing 2% FBS and 0.25% Fc Receptor Binding Inhibitor Functional Grade Monoclonal Antibody for 10 min at room temperature. Subsequently, cells were stained with intracellular markers overnight on a rotating wheel (3 rpm) at 4 °C and protected from light. Finally, cells were washed three times with 2% FBS and resuspended in 2% FBS containing 0.5 g/mL DAPI. All samples were measured on a BD Bioscience LSR2 upgraded flow cytometer, and 10,000 events were recorded. FlowJo V10 (Tree Star, Inc. (Ashland, OR, USA, FlowJo, RRID: SCR_008520)) was used to analyze FCS files. All antibodies are listed in Supplementary Table S1. For the ALDEFLUOR (Cat. #01700; STEMCELL Technologies), staining was performed according to the manufacturer's protocol (https://cdn.stemcell.com/media/files/pis/29888-PIS_1_1_2.pdf and https://www.biovision.com/documentation/datasheets/K936.pdf), then measured and analyzed as above. An inhibitor of ALDH activity, *N*,*N*-diethylaminobenzaldehyde (DEAB), was used as a negative control for this assay. Staining for mitochondrial mass was performed as described before [65].

### Cell viability assay

Scramble control and siLDHB cells were plated at 3000 cells/well in 96-well plates for overnight culture. For the lactate rescue experiment, cells were changed into DMEM no glucose medium (cat #11966025; Thermo Scientific) containing 20 mM L-lactic acid (cat #27714; Sigma-Aldrich) or HCl and adjusted to pH 6.8. For the nucleotide rescue experiment, different concentrations of hypoxanthine, adenine, and uridine or solvents were added to the cells in DMEM/F12 medium. After 4 days, cell viability was determined by Acid Phosphatase (APH) Assay according to the protocol described previously [66]. Absorbance was quantified in a Tecan Infinite^®^ M1000 Microplate Reader.

### Immunoblotting

Cell lysates were extracted in 1X RIPA Lysis and Extraction Buffer (Cat. #89901; Thermo Fisher Scientific) with 2X Protease and Phosphatase Inhibitor Cocktail (Cat. #78440; Thermo Fisher Scientific) for 20 min on ice. The lysate was purified by centrifugation at 14,000 *g* for 25 min at 4 °C. Protein concentration was quantified using the BCA Protein Assay Kit (Cat. #23209; Thermo Fisher Scientific). Samples were resolved by SDS–PAGE and then transferred using Trans-Blot^®^ Turbo™ Mini Nitrocellulose Transfer Packs (Cat. #1704158; Bio-Rad). Prior to staining with antibodies, membranes were blocked with TBS (Cat. #927-60001; LI-COR Biosciences) for 1 h at room temperature. Subsequently, the membranes were incubated with the primary antibodies overnight on a rotating wheel (3 rpm) at 4 °C. After washing three times with TBS wash buffer (Tris Buffered Saline (1 tablet/500 mL; Cat. #94158-10TAB; Sigma Aldrich Chemie GmbH) + 2% TWEEN 20(Cat. #P1379; Sigma-Aldrich)), the membranes were incubated with secondary antibodies of IRDye 680LT-conjugated goat anti-mouse IgG (1: 5000; (LI-CORBiosciences Cat# 926-68020, RRID:AB_10706161), and IRDye 800CW-conjugated goat anti-rabbit IgG (1:5,000; LI-COR Biosciences Cat# 926-32211, RRID:AB_621843) were incubated for 30 min at room temperature. All primary antibodies are listed in Supplementary Table S1. Images were acquired and analyzed using the Odyssey Infrared Imaging System (Li-COR Biosciences).

### Immunofluorescence microscopy

Cells grew on 4-well chamber slides (cat. #154526; Thermo Scientific Nunc) and reached 50–80% confluency. Cells were then fixed with 4% paraformaldehyde for 20 min at RT and permeabilized with 0.2% Triton X-100 for 15 min. The cells were then treated with acetone and methanol (1:1) for 20 min at room temperature. After the cells were blocked with 1% BSA for 2 h at room temperature, the cells were stained with the antibodies listed in Supplementary Table S1. Then cells were mounted by mount buffer containing DAPI (Cat. # P-3693; Thermo Fisher Scientific). For mitochondrial mass staining, cells were pretreated with 200 nM MitoTracker^®^ Deep Red FM (Cat. #M22426; Thermo Fisher Scientific) for 1 h in a cell culture incubator. Then procedures were continued as described above. Images were acquired by ZEISS_LSM 710 confocal microscope and processed by Fiji.

### Immunohistochemistry

Immunohistochemistry was performed at room temperature using the fully automated BOND RX^®^ staining system (Leica Biosystems) as previously described [67]. Samples were stained with appropriate antibodies (Supplementary Table S1). Images were acquired and processed using Fiji.

### LDHB activity

LDHB activity was measured by the LDH-B Activity Assay kit (Cat. #ab140361; Abcam) according to the manufacturer’s instructions.

### RNA Sequencing and data analysis

After gene silencing by siRNA, RNA sequencing was performed from A549 (NCI-DTP Cat# A549, RRID:CVCL_0023) and H358 (NCI-DTP Cat# NCI-H358, RRID:CVCL_1559) cell cultures. Total RNA was isolated and purified with RNeasy Mini Kit (Cat. #74106, Qiagen). The quantity and quality of the purified total RNA were assessed using a Thermo Fisher Scientific Qubit 4.0 fluorometer with the Qubit RNA BR Assay Kit (Cat. #Q10211; Thermo Fisher Scientific) and an Advanced Analytical Fragment Analyzer System using a Fragment Analyzer RNA Kit (Cat. #DNF-471; Agilent), respectively. Sequencing libraries were made using an illumina TruSeq Stranded mRNA Library Prep kit (Cat. #20020595; illumina) combined with TruSeq RNA UD Indexes (Cat. #20022371; illumina) according to illumina’s guidelines. Pooled cDNA libraries were sequenced paired-end using an illumina NovaSeq 6000 SP Reagent Kit (Cat. #20028401, 100 cycles; illumina) on an illumina NovaSeq 6000 instrument. The run produced, on average, 41 million reads/sample. The quality of the sequencing run was assessed using illumina Sequencing Analysis Viewer (illumina version 2.4.7) and all base call files were demultiplexed and converted into FASTQ files using illumina bcl2fastq conversion software v2.20. The quality control assessments, generation of libraries, and sequencing were conducted by the Next Generation Sequencing Platform, University of Bern.

Pathway enrichment analysis was performed using Metascape (RRID:SCR_016620), and the Gene Set Enrichment Analysis (GSEA) was performed in GSEA software (SeqGSEA, RRID:SCR_005724) [68–70]. The gene set of Mitochondrial respiratory chain complexes was acquired from HUGO Gene Nomenclature Committee and analyzed with GSEA using R 4.0.2.

### Gene silencing by small interfering (siRNA) and short hairpin RNAs (shRNA)

For transient knockdown, cells were cultured in 6-well plates overnight until 50–70% confluence was achieved. Cells were then transfected with Lipofectamine 2000 (Cat. #11668027; Invitrogen) and pooled LDHB human siRNA Oligo Duplex (Cat. #SR320835; Origene) according to the manufacturer’s protocol. The Trilencer-27 Universal Scrambled Negative Control siRNA Duplex was used as a negative control.

For stable knockdown, the LDHB Human shRNA Plasmid Kit (Cat. #TL311768; Origene) was used to produce lentivirus according to The RNAi Consortium (TRC) Broad Institute protocol. The shRNA sequences are listed as follows: Control shRNA: Sense Target strand GCACTACCAGAGCTAACTCAGATAGTACT, Loop TCAAGAG, Antisense Target strand AGTACTATCTGAGTTAGCTCTGGTAGTGC; shLDHB-1 Sense Target strand TGAATGTGGCAGGTGTTTCTCTCCAGGAA, Loop TCAAGAG, Antisense Target strand TTCCTGGAGAGAAACACCTGCCACATTCA; shLDHB-2 Sense Target strand AGTCTCTGGCTGATGAACTTGCTCTTGTG, Loop TCAAGAG, Antisense Target strand CACAAGAGCAAGTTCATCAGCCAGAGACT. Cells were infected with lentivirus to generate stable LDHB knockdown or scramble control cell lines according to the protocol from Addgene (https://www.addgene.org/protocols/generating-stable-cell-lines/). After infection, cells were selected after 3 days of treatment with 2 μg/mL puromycin. The cells selected with puromycin were then sorted using the GFP marker by flow cytometer. Subsequently, 100 cells were plated in 150mmX20mm cell culture dishes (cat #20151; Bioswisstec) treated with 2 μg/mL puromycin. Isolated colonies were identified microscopically. The medium was aspirated and washed with PBS. 8-mm cloning cylinders (cat. #TR-1004; MERCK Millipore) were placed around individual clones and filled with 200 µL of 1 × TrypLE (cat. #A1217702; Thermo Fisher Scientific). After incubation at 37 °C for 5 min, detached cells were transferred to tissue culture-treated 6-well plates (Cat. #174932; Thermo Fisher Scientific) and cultured to 80–90% confluence for 6 days. Individual clones were verified by Western blot. All stable clones were used for experiments within passages 4–8.

### Mitochondrial complex activity

Two million cells were harvested and resuspended in a mitochondrial respiration medium prepared according to the protocol from Oroboros (https://www.bioblast.at/images/d/d9/MiPNet14.13_Medium-MiR06.pdf). Mitochondrial complex I, II, IV activity is quantified using Oroboros O2k (Bioblast, Austria) according to the previously described protocol [71]. All chemicals are listed in Supplementary Table S2. The data were acquired and processed with DatLab4.0 (Bioblat).

### Quantitative real-time PCR (qRT-PCR)

Total cell DNA was extracted and purified using GenElute™ Mammalian Genomic DNA Miniprep Kit (Cat. #G1N350; Sigma). DNA concentration was quantified by Nanodrop 2000 (Thermo Fisher Scientific). A protocol published previously [72] has been adapted to measure DNA damage by real-time PCR (rtPCR). The method is based on using a DNA polymerase capable of generating long DNA fragments (LRPCR), a process blocked by DNA damage. Thus, target genomic DNA containing lesions will be less amplified compared to a non-damaged control genomic DNA. In this method, 3 different gene fragments are targeted: β-globin (13 kb, 5′ region, non-transcribed), Hprt (10.4 kb, transcribed), and Mito (8.9 kb, lack of histones). All primers were synthesized and ordered from Microsynth, see Supplementary Table S3. The qRT-PCR was performed in 96-well plates. For SRPCR, 10 μL of reaction mix for each well contained 15 ng of DNA, 1 μM β-globin primer or 1 μM Mito DNA primer and 5 μL GoTaq@ qPCR master mix (2x) (Cat. #A6002; Promega), 2 μL nuclease-free water. Run method was as follows: Hold stage 95.0 °C 2 min; cycling stage Step1 95.0 °C, 3 s, Step2 68.0 °C 30 s, 40 cycles; melt curve stage using a continuous method Step1 95.0 °C 15 s, Step2 50.0 °C 1 min, Step3 95.0 °C 15 s, Step4 60.0 °C 15 s. For LRPCR, 20 μL of reaction mix for each well contained 30 ng of DNA, 0.2 μM β-globin primer or 1 μM Mito DNA primer or 1 μM Hprt primer, and 10 μL GoTaq@ Long qPCR Master Mix (Cat. #M4021; Promega), 2 μL 1:4 diluted EvaGreen® Dye 20X in water (cat. #31000; Biotium), 0. 2 μL CXR reference dye (cat. #C5411; Promega), 1.8 μL nuclease-free water. Run method was as follows: Hold stage 95.0 °C 2 min; cycling stage Step1 92.0 °C, 30 s, Step2 68.0 °C 15 min, 40 cycles; melt curve stage using a continuous method Step1 95.0 °C 15 s, Step2 50.0 °C 15 s, Step3 95.0 °C 15 s, Step4 60.0 °C 15 s. The reactions were performed in a 7500 Fast Real-Time PCR System (Thermo Fisher Scientific), and samples were measured in triplicates. Using the Δ2CT method, the expression of LRPCR fragments is normalized to the expression of a small genomic DNA fragment (258 bp) for β-globin and Hprt, and a small mitochondrial DNA fragment (221 bp) for Mito using regular short-range PCR (SRPCR).

### NMR

NMR experiments were performed on a 500.13 MHz Bruker Avance II spectrometer (Bruker BioSpin) equipped with a 5 mm ATM BBFO probe with a z-gradient. 13C NMR spectra were acquired using a 1D sequence with inverse gated decoupling and a flip angle of 30° (“zgig30” from the Bruker pulse program library). Each 1D 13C NMR spectrum was measured using the same parameters: a nominal temperature of 275 K, a spectral width of 34,000 Hz, a data size of 32 K points, 1280 transients, an acquisition time of 0.48 s, and a relaxation delay of 4 s. The total experiment time for the 13C NMR acquisition was about 96 min. The spectral processing was performed using the Bruker Topspin software (version 3.2, patch level 5). The free induction decays (FIDs) were exponentially weighted with a line broadening factor of 10 Hz, Fourier-transformed, manually phased, and baseline corrected. For each sample, the 13C lactate peak of interest was integrated. The absolute integral was then converted into an mM value using the integral of the starting condition with a known concentration of 20 mM lactate. For the analysis, lactate consumption values, normalized for the cell number, were calculated via subtraction of the lactate concentration value of a specific sample from 20 mM lactate at the starting condition.

### LC–MS

5 × 10^5^ scramble control cells and siLDHB cells were plated out in 6-well plates in DEME/F12 medium. After 48 h, the medium was aspirated and then carefully washed with 2 mL of prewarmed (37 °C) wash solvent (75 mM ammonium carbonate, pH was adjusted to 7.4 with acetic acid). The wash solvent was completely aspirated. 400 μL of the pre-cooled (− 20 °C) extraction solvent (40% acetonitrile, 40% methanol, 20% nanopure water) was immediately added to the plates. All chemicals are listed in Supplementary Table S2. Plates were sealed with aluminum adhesive foil and immediately placed at − 20 °C. After 1 h, plates were transferred to the freezer at − 80 °C. The metabolites were extracted by scraping the surface of the plates with a cell scraper on ice. Transfer the extract to a 2 mL Eppendorf tube and spin the tube at 5000 g for 5 min at 4 °C. The clean supernatants were transferred into 2 mL Eppendorf tubes and stored immediately at − 80 °C for further analysis by LC–MS measurements. LC–MS measurement and analysis were described previously [73].

### In vivo limiting dilution assay

The mouse experiments were performed in accordance with the animal welfare guidelines and protocols approved by the Institutional Animal Care and Ethical Committee; license number BE 8/16. Cells were cultured in vitro for 6 days before transplantation. Various tumor cell suspensions were inoculated subcutaneously (left and right flank) in 100 μL serum-free medium and growth factor-reduced Matrigel (1:1) (Cat. #356231, Corning) at limiting dilutions (10,000, 1,000, 100, 10). Age- and sex-matched NOD-scid IL2Rγnull (NSG) mice maintained under specific pathogen-free conditions were used as recipients. Tumors were explanted after 6 weeks. Extreme limiting dilution analysis (ELDA) was performed as described [74].

### Genetically engineered mouse model

The LDHB deficient sperms were ordered from the International Mouse Strain Resource (IMSR) platform, and oocytes were obtained from superovulation mature female KP mice, which were used to perform in vitro fertilization (IVF). Subsequently, the embryos were transferred to recipient females. The genotype of all puppies was tested by Southern blot. To generate LDHB^+/+^; K-ras^LSL−G12D/+^; p53^fl/fl^ and LDHB^−/−^; K-ras^LSL−G12D/+^; p53^fl/fl^, 2.5 × 10^7^ PFU AAV-Cre virus in a total volume of 50 μL was introduced to 8-week-old LDHB^+/+^; K-ras^LSL−G12D/+^; p53^fl/fl^, LDHB^±^; K-ras^LSL−G12D/+^; p53^fl/fl^ LDHB^−/−^; K-ras^LSL−G12D/+^; p53^fl/fl^ mice after anesthesia. Two weeks later, all mice were scanned with microCT (X-RAD SmART–Precision X-Ray) to determine the basal line for the lung. Subsequently, mice were scanned with microCT every 2 weeks to assess tumor development and sacrificed at 16–18 weeks of age. The microCT images were processed and analyzed with Fiji and 3D Slicer version 4.13 according to a previously published protocol [75, 76].

### Human PCTS

The experiments with human patient samples were performed in accordance with the Declaration of Helsinki and protocols approved by the local ethics committee of the Canton of Bern; license number PB_2016-01560. In detail, NSCLC tissue specimens were acquired in collaboration with the Tissue Bank Bern. Patients of both sexes were included; age and weight were not matched. A solution of 4% (w/w) low gelling agarose (Cat. #A9414-100G; Sigma-Aldrich) in DMEM cell culture medium was prepared and heated in a microwave (700 W) for a few seconds until obtaining a homogenous mix. The solution was then cooled down and kept at 37–38 °C, ready to use. Meanwhile, the NSLC tumors resected from patients were manually cleaned from any surrounding residual lung parenchyma and subsequently cut into regular cubes with an edge length of approximately 1 cm. The cubes were embedded in the low gelling agarose solution that solidified on ice for about 15/20 min. The agarose-embedded piece of the tumor is transferred to a cutting apparatus, which was in-house designed and made, resulting in PCTS of 500 µm-thickness. The slices, of irregular shapes, were homogenized using a round tissue punch of 6 mm diameter. The slices were plated in 6-well plates (one slice per well), and 1750 µL of medium were added per well. PCTS were cultured at 37 °C with 5% CO_2_. Viability of the slices with complete Medium (DMEM 4.5 g/L d-Glucose, Cat. #41966-052; Gibco) was assessed up to 4 day post-incubation. Slices (in duplicates) were cultured with four different medium conditions: condition 1, 2.5 mM glucose DMEM Medium with no lactate; condition 2, 2.5 mM glucose DMEM medium with 20 mM lactate; condition 3, 10 mM glucose DMEM medium without lactate; condition 4, 2.5 mM glucose DMEM Medium with 20 mM lactate.

Ex vivo imaging of PCTS was performed 3 day post-incubation at a confocal microscope Zeiss LSM 880. Slices were removed from the cell culture medium, quickly washed twice in Ringer’s solution, and stained for 30 min with the kit LIVE/DEAD™ Viability/Cytotoxicity Kit, for mammalian cells (Cat. #L3224; Thermofisher) and HOECHST suspended in a solution of HEPES 0.01 M (Cat. #15630-049; Gibco) and Vitamin C 140 mM in Ringer’s. At the end of the incubation, slices were washed twice and mounted on a MatTek glass-bottom microwell dish (35 mm No. 0) for live imaging. The whole slices were imaged in tile scan mode, and stitching processing was then applied. Maximum Intensity Projection of the acquired z-stacks was obtained and finally used for image analysis and image visualization. The complete experiment was repeated twice, and the analysis was randomized.

All images were analyzed using Fiji with the same brightness, contrast, and threshold settings for each channel and experiment. Calcein AM staining (green), EthD-1 staining (red), and HOECHST staining (blue) signals were area determined and restricted to the tissue sample corresponding to the TL (bright field) channel.

### Public data source and analysis

Analysis of LDHB gene expression at different stages of lung cancer development in the mouse model was obtained from the GSE13963 data set https://www.ncbi.nlm.nih.gov/geo/query/acc.cgi?acc=GSE13963. LDHB gene expression in normal patient tissues and LUAD patients with TP53 mutation or TP53 non-mutation was analyzed using UALCAN based on the TCGA database (http://ualcan.path.uab.edu/analysis.html). LDHB expression in LUAD, LUSC tumors, and matched normal tissues were analyzed by GEPIA2 based on the TCGA database (http://gepia2.cancer-pku.cn/#index). Overall survival data and analysis were obtained and analyzed using the Kaplan–Meier plotter (Kaplan–Meier plotter [Lung] (kmplot.com)).

### Statistical analysis

Statistical analysis was performed using GraphPad Prism 9 (GraphPad Prism, RRID: SCR_002798). Repeated measurements were made on different biological samples. Pearson R was reported for correlation analysis.  Error bars represent mean ± standard deviation (SD) or mean ± standard error of the mean (SEM). Ordinary one-way ANOVA and two-way ANOVA were followed with Tukey’s multiple comparison test. Two-tailed unpaired or paired Student’s *t*-tests were performed, as described in figure legends. The *P* values < 0.05 were considered significant. In all analyses, the significance level is presented as follows: *P < 0.05, **P < 0.01, ***P < 0.001, ****P < 0.0001*.

### Supplementary Information

Below is the link to the electronic supplementary material.

Supplementary file1 Supplementary Fig. S1: a. Gene expression of selected genes in HOLO and PARA clones (n=3). *P < 0.05 (two-tailed unpaired Student’s t-test). b-c. Basal, ATP-linked, maximal, and spare oxygen consumption rate (OCR) of HOLO and PARA clones were measured as described in Fig.1. The OCR was normalized to mean OCR of HOLO cells. The error bars represent mean ± SD (n=3 biological replicates). ****P < 0.0001 (two-tailed unpaired Student’s t-test). d. The glycolytic function is represented by glycolysis, glycolytic capacity, and reserve glycolysis. The ECAR was normalized to mean ECAR of HOLO cells. The ECAR fold change to HOLO cells was shown by bar graph (n=4 biological replicates). The error bar was represented with mean ± SD. ***P < 0.001, ****P < 0.0001 (Ordinary two-way ANOVA). e. Flow cytometer plot with ALDEFLUOR (ALDH) or GLDC by using FlowJo for A549 and H358 cell lines, which were cultured in 20 mM L-lactic acid or HCl and adjusted to pH 6.8 as described in Fig.1. Supplementary Fig. S2: a. Immunoblot analysis of H358, H460, H838, and PF139 cells transfected with control siRNA (siCTRL) or LDHB-specific siRNA (siLDHB) (10 nM) using Lipofectamine 2000 after 48 hours. β-actin was used as the loading control. b-c. The oxygen consumption rate (OCR) of siCTRL and siLDHB cells were measured as described in Fig.1. The ATP-linked and maximal OCR of siLDHB cells were normalized to corresponding siCTRL and plotted as bar graphs (n=3-4 biological replicates). The error bars represent mean ± SD. ****P < 0.0001 (two-tailed unpaired Student’s t-test). d. Left panel: Representative plot showing mean ± SEM of the real-time extracellular acidification rate (ECAR) across treatments after 48 hours of transfection with siCTRL or siLDHB using the Seahorse XFe96 analyzer (n=23 technical replicates with 3-5 readings). Right panel: Extracellular acidification rate (ECAR) of siLDHB cells from Mito Stress Test was normalized to corresponding siCTRL and plotted as bar graphs (n=3-4 biological replicates). The error bars represent mean ± SD. ****P < 0.0001 (two-tailed unpaired Student’s t-test). e. The glycolytic function is represented by glycolysis, glycolytic capacity, and reserve glycolysis. The ECAR was normalized to mean ECAR of A549 siCTRL cells. The ECAR fold change to A549 siLDHB cells was shown by bar graph (n=4 biological replicates). The error bar was represented with mean ± SD. **P < 0.01, ****P < 0.0001 (Ordinary two-way ANOVA). f. The energy status for siCTRL and siLDHB was visualized by plotting the basal OCR against the basal ECAR from one representative experiment (n=10-40 technical replicates). g. Extracellular acidification rate (ECAR) of shRNAs cells from Mito Stress Test were analyzed (n= 4 biological replicates). The error bar was represented with mean ± SD. *P < 0.05, **P < 0.01, ***P < 0.001 (Ordinary one-way ANOVA). Supplementary Fig. S3: a. Statistical analysis of PARA population based on flow cytometer results in Fig.3a. (n=3). *P < 0.05 (two-tailed unpaired Student’s t-test). b. 1.0 x 10^5^ siCTRL or siLDHB HOLO or PARA cells were seeded into 6-well plates, and cell numbers were determined after 3 days. In the right panel, the cell number of siLDHB HOLO and PARA was normalized to the corresponding siCTRL and analyzed (n=5). **P < 0.01 (two-tailed unpaired Student’s t-test). c. Analysis of the apoptosis of HOLO cells by flow cytometer using Annexin V and PI staining co-staining after 48 hours of transfection with siCTRL or siLDHB. The early and late apoptotic population was shown by Annexin V+/PI− and Annexin V+/PI+, respectively. Analysis of HOLO by flow cytometer using EpCAM and CD90 co-staining after 48 hours of transfection with siCTRL or siLDHB. The epithelial-like stem cell population and meroclonal cell population was represented by EpCAM+CD90- and EpCAM-CD90- respectively (n=3). ***P < 0.001 (two-tailed unpaired Student’s t-test). Error bars represent mean ± SD. d-e. GLDC expression or ALDEFLUOR (ALDH) activity of the H358 and H460 cell lines was determined by flow cytometry. f. 500 siCTRL or siLDHB cells were seeded in Nunclon Sphera 6-well plates and cultured with 3D CnT culture medium or 2D DMEM/F12 or RPMI medium after transfection with siCTRL or siLDHB. For the sphere formation assay, spheres were counted under the microscope, and for the colony formation assay, colonies were counted with Fiji (n=3) after 7-21 days. Images were taken after 7-14 days. *P < 0.05, **P < 0.01, ***P < 0.001 (two-tailed unpaired Student’s t-test). Data are shown with mean ± SD. g. LDHB gene expression in normal patient tissues and LUAD patients with TP53 mutation or TP53 non-mutation was analyzed using UALCAN based on the TCGA database. h. Flow cytometer plot showing HOLO and PARA populations, and GLDC positive population in A549 shCTRL and shLDHB cells. i. Representative images showing spheres and colonies from the sphere formation and colony formation assay as described above. j. Left panel: Immunohistochemical staining of xenograft tumors from A549 shCTRL and shLDHB clones with LDHB and GLDC staining. Scale bars indicate 100 μm. Right panel: Raw data for ELDA analysis. k. 1.0 x 10^5^ siCTRL or siLDHB cells were seeded into 6-well plates, and cell numbers were determined after 3 days (n=3). ns no significant difference, *P < 0.05, **P < 0.01 (two-tailed unpaired Student’s t-test). Data are shown with mean ± SD. l. Analysis of the effect of siLDHB on the normal cell line BEAS-2B by proliferation assay, cell viability assay, and colony formation assay as previously described. Analysis of protein expression by immunoblot as previously described. Supplementary Fig. S4: a. Colocalization analysis of mitochondria and LDHB protein by staining mitochondria with MitoTracker® Deep Red FM (red) and LDHB with Alexa Fluor 488 conjugated antibody (green). The images were acquired by a confocal microscope and analyzed by Fiji. b. Volcano plot showing the enrichment of gene sets in siCTRL and siLDHB cells of A549 and H358. The gene sets in the upper left and right quadrants are significantly enriched in siCTRL and siLDHB, respectively. NES Normalized Enrichment Score. c. Maximal ADP-stimulated respiration was quantified by OROBOROS to evaluate mitochondrial respiration complex I (CI), II (CII), IV (CIV) activity in A549 siCTRL and A548 siLDHB after 48 hours of transfection or A549 shCTRL and shLDHB cells after supplementation with digitonin (permeabilization), glutamate and malate (activation of complex I activity), rotenone (complex I inhibitor), succinate (activation of complex II activity), antimycin A (complex II inhibitor), ascorbate/TMPD (activation of complex IV activity), NaN3 (complex IV inhibitor) sequentially. d. Gene expression of selected genes in A549 siCTRL and A549 siLDHB cells (n=3). The error bar was represented with mean ± SD. ns no significant difference, **P < 0.01, ***P < 0.001 (two-tailed unpaired Student’s t-test). Immunoblot analysis of A549 siCTRL and A548 siLDHB after 48 hours using Total OXPHOS Human WB Antibody Cocktail. β-actin was used as the loading control. e. The NAD+ and NADH of A549 shCTRL and shLDHB cells were quantified using an enzymatic colorimetric kit (n=3). ***P < 0.001, ****P < 0.0001 (Ordinary two-way ANOVA). The error bar was represented with mean ± SD. Supplementary Fig. S5: a. Balloon plot showing metabolite enrichment analysis (MSEA) of upregulated metabolites in A549 and H358 after LDHB silencing. The size and color of the balloon indicate the enrichment ratio and P-value, respectively. b-c. Combination analysis of whole-genome sequencing and metabolomics data to show TCA cycle-related genes and intermediates. Red *: significantly decreased in H358 only; Blue *: significantly increased in A549 only. Otherwise decreased in both of A549 and H358. The log2 fold change in amino acids was presented. A two-tailed unpaired T-test was used for the statistical analysis (n=3 biological replicates with 2 technical replicates). *P < 0.05, **P < 0.01, ***P < 0.001, ****P < 0.0001. d. Analysis of the intensity of pyrimidine-, purine-, and TCA cycle-related metabolites. (n=3 biological replicates with 2 technical replicates). *P < 0.05, **P < 0.01, ***P < 0.001, ****P < 0.0001 (two-tailed unpaired Student’s t-test). e. Heatmap showing the metabolomic comparison of NOMO1 cells treated with PBS or R-2HG from Qing's publication. Three replicates are shown as separate columns for each cell type. Venn diagram shows the number of dysregulated metabolites in A549 cells after LDHB silencing and NOMO1 cells after R-2HG treatment (Qing et al.). Balloon plot showing significantly downregulated metabolic pathways in both A549 siLDHB cells and NOMO1 cells after R-HG treatment. f. A549 siCTRL or siLDHB cells were rescued by the addition of nucleotide precursors or corresponding solvents. Concentration was titrated based on cell viability as follows: Conc. 1: 50µM hypoxanthine, 50µM adenine, 200µM uridine; Conc. 2: 100µM hypoxanthine, 100µM adenine, 400µM uridine; Conc. 3: 200µM hypoxanthine, 200µM adenine, 800µM uridine; 400µM hypoxanthine, 400µM adenine, 1600µM uridine. Finally, Con.2 was used for the further rescue experiment. Supplementary Fig. S6: a. LDHB expression in LUAD, LUSC tumors, and matched normal tissues were analyzed by GEPIA2 based on the TCGA database. b. Kaplan–Meier plots of overall survival of lung adenocarcinoma (LUAD) and lung squamous cell carcinoma (LUSC) based on LDHB mRNA expression. c. PCTS was cultured in 10 mM glucose DMEM medium with or without 20 mM sodium lactate and subsequently stained with Calcein AM, EthD-1, and HOECHST after 3 days. The images were taken with a Zeiss LSM 880 confocal microscope and analyzed by Fiji software. The Live/Dead ratio is presented (n=2). **P < 0.01 (two-tailed paired Student’s t-test). Supplementary Fig. S7: a. Southern blot analysis of mice tissue for LDHB^+/+^; K-ras^LSL-G12D/+^; p53^fl/fl^, LDHB^+/-^; K-ras^LSL-G12D/+^; p53^fl/fl^, and LDHB^-/-^; K-ras^LSL-G12D/+^; p53^fl/fl^ mice. The picture shows the ladder of the southern blot and the table shows the size of different markers. b. LDHB gene expression at different stages of lung cancer development in the GSE13963 dataset. c. Representative axial CT scans from additional LDHB^+/+^; K-ras^LSL-G12D/+^; p53^fl/fl^ and LDHB^-/-^; K-ras^LSL-G12D/+^; p53^fl/fl^ mice after intratracheal instillation with AAV-Cre virus at different time points. (PDF 3234 KB)

Supplementary file2 (PDF 139 KB)

Supplementary file3 (XLSX 110 KB)

Supplementary file4 (XLSX 14005 KB)

Supplementary file5 (XLSX 3677 KB)

## Data Availability

All data generated or analyzed during this study are included in this published article and its supplementary information files.

## Abbreviations

LDH: Lactate dehydrogenase
LDHA: Lactate dehydrogenase A
LDHB: Lactate dehydrogenase B
NSCLC: Non-small-cell lung cancers
TICs: Tumor-initiating cells
GLDC: Glycine decarboxylase
ELDA: Extreme limiting dilution analysis
HK2: Hexokinase 2
ALDH: Aldehyde dehydrogenase
DEAB: *N*,*N*-Diethylaminobenzaldehyde
mtDNA: Mitochondrial DNA
ETC: Electron transport chain
GSEA: Gene set enrichment analysis
ROS: Reactive oxygen species
OCR: Oxygen consumption rate
ECAR: Extracellular acidification rate
HOLO: Holoclonal A549 cells
PARA: Paraclonal A549 cells
