# Supplementary material for: Targeting lactate dehydrogenase B-dependent mitochondrial metabolism affects tumor initiating cells and inhibits tumorigenesis of non-small cell lung cancer by inducing mtDNA damage
Source: Cell Mol Life Sci. 2022 Jul 25;79(8):445. doi: 10.1007/s00018-022-04453-5 (PMC9314287; doi:10.1007/s00018-022-04453-5)

Fig. S1

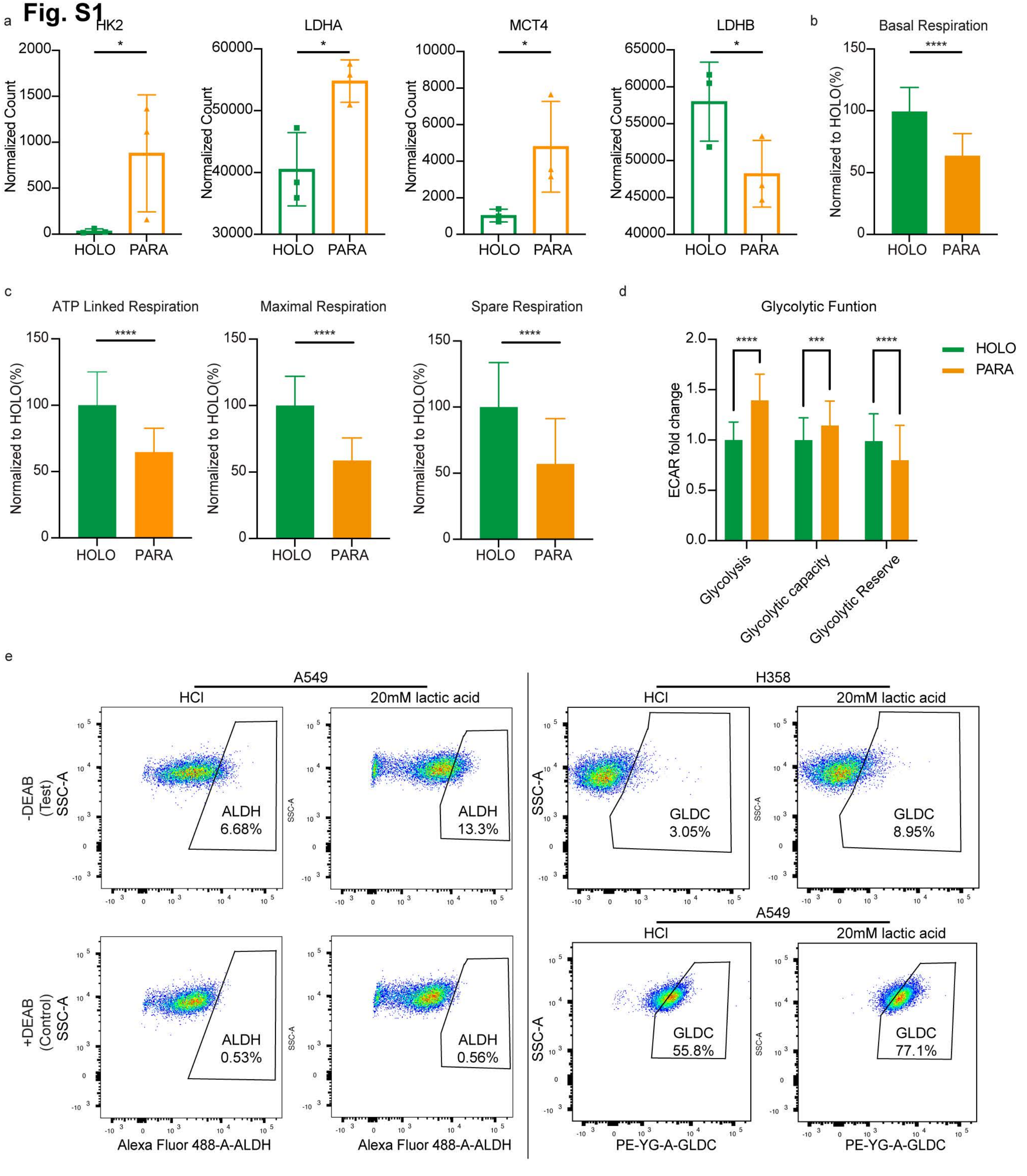

a
HK2
LDHA
MCT4
LDHB
Normalized Count
HOLO
PARA
b
Basal Respiration
Normalized to HOLO(%)
c
ATP Linked Respiration
Maximal Respiration
Spare Respiration
d
Glycolytic Funtion
ECAR fold change
Glycolysis
Glycolytic capacity
Glycolytic Reserve
e
A549
H358
HCl
20mM lactic acid
-DEAB (Test) SSC-A
+DEAB (Control) SSC-A
ALDH 6.68%
ALDH 13.3%
ALDH 0.53%
ALDH 0.56%
GLDC 3.05%
GLDC 8.95%
GLDC 55.8%
GLDC 77.1%
Alexa Fluor 488-A-ALDH
PE-YG-A-GLDC

Fig. S2

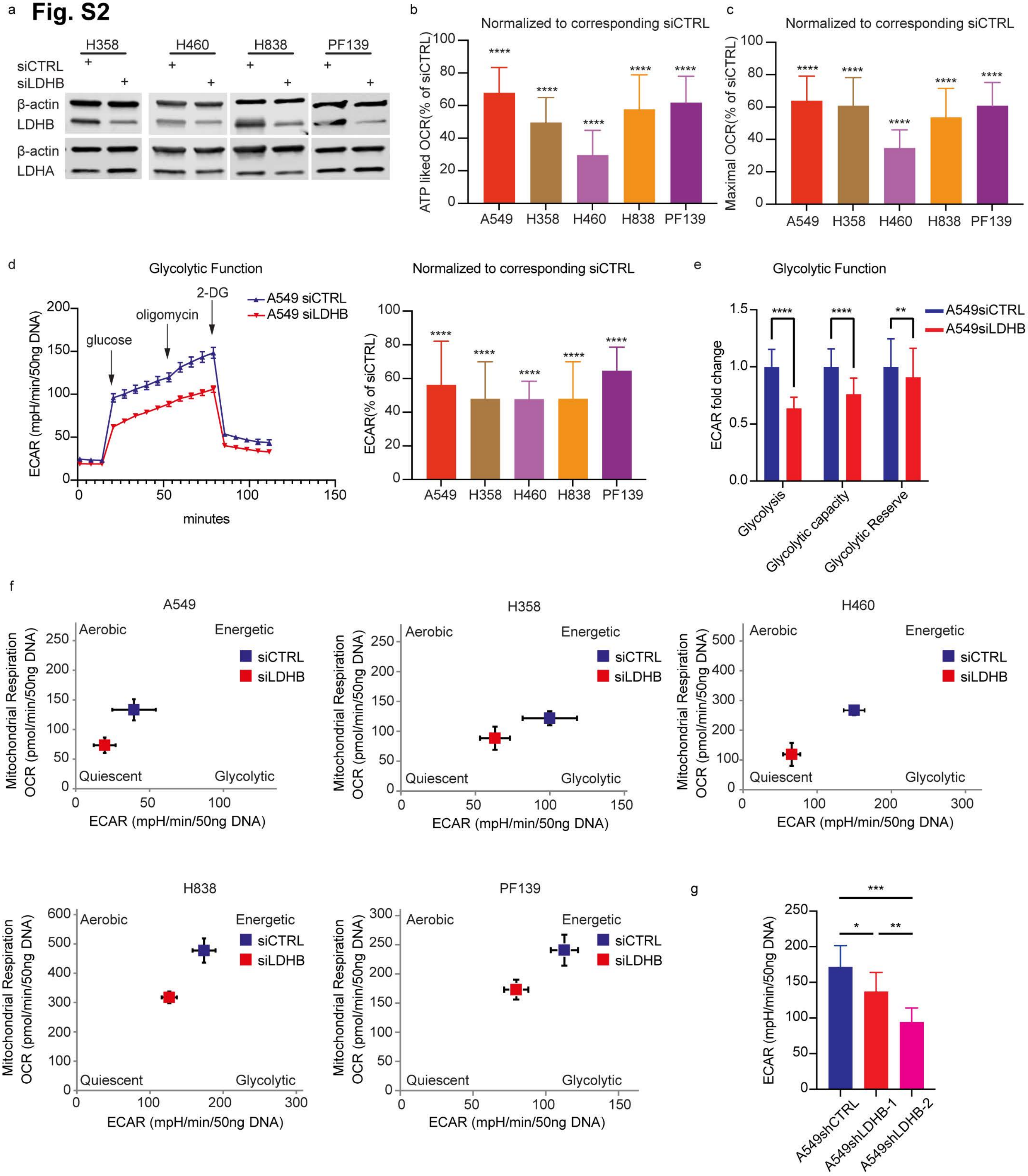
a
H358
H460
H838
PF139
siCTRL
siLDHB
β-actin
LDHB
β-actin
LDHA
b
Normalized to corresponding siCTRL
ATP liked OCR(% of siCTRL)
A549
H358
H460
H838
PF139
c
Normalized to corresponding siCTRL
Maximal OCR(% of siCTRL)
d
Glycolytic Function
ECAR (mpH/min/50ng DNA)
glucose
oligomycin
2-DG
A549 siCTRL
A549 siLDHB
minutes
Normalized to corresponding siCTRL
ECAR(% of siCTRL)
e
Glycolytic Function
ECAR fold change
A549siCTRL
A549siLDHB
Glycolysis
Glycolytic capacity
Glycolytic Reserve
f
A549
H358
H460
H838
PF139
Aerobic
Energetic
Quiescent
Glycolytic
siCTRL
siLDHB
Mitochondrial Respiration
OCR (pmol/min/50ng DNA)
ECAR (mpH/min/50ng DNA)
g
ECAR (mpH/min/50ng DNA)
A549shCTRL
A549shLDHB-1
A549shLDHB-2

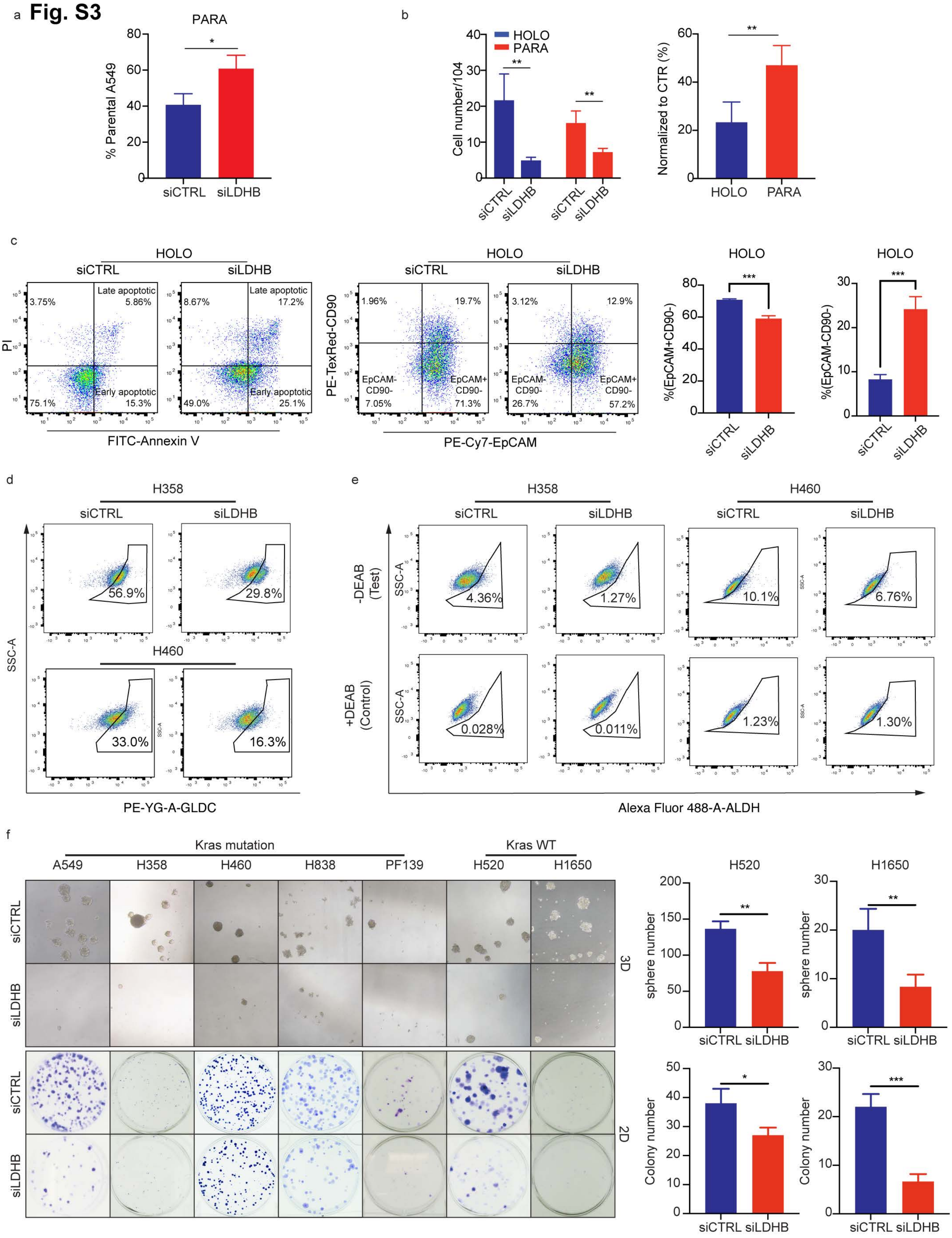

Fig. S3
a
PARA
% Parental A549
siCTRL
siLDHB
b
HOLO
PARA
Cell number/104
Normalized to CTR (%)
c
HOLO
siCTRL
siLDHB
Late apoptotic
Early apoptotic
PI
FITC-Annexin V
PE-TexRed-CD90
PE-Cy7-EpCAM
EpCAM-CD90-
EpCAM+CD90-
%(EpCAM+CD90-)
%(EpCAM-CD90-)
d
H358
H460
SSC-A
PE-YG-A-GLDC
e
-DEAB (Test)
+DEAB (Control)
Alexa Fluor 488-A-ALDH
f
Kras mutation
Kras WT
A549
H358
H460
H838
PF139
H520
H1650
3D
2D
sphere number
Colony number

Fig. S3

g

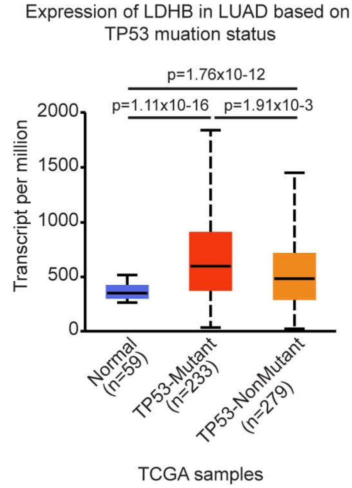

h

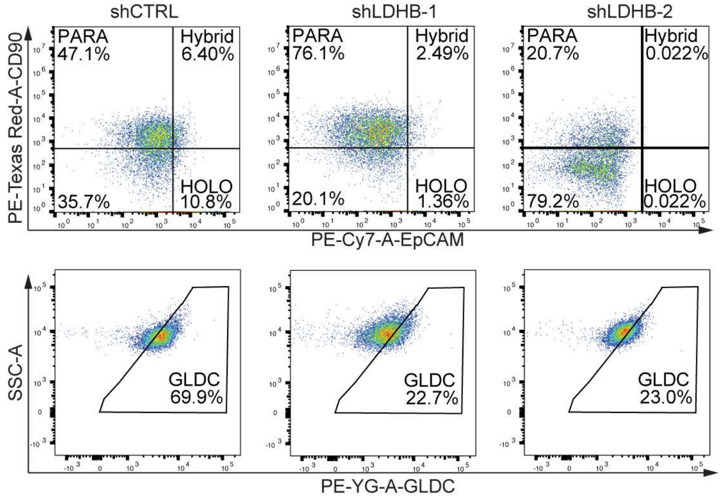

i

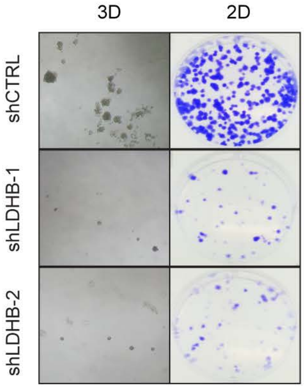

j

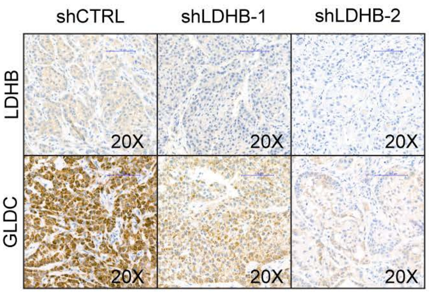

Limiting dilution data entered

Counter	Dose	Tested	Response	Group
1	10	4	2	A549 shCTRL
2	100	4	3	A549 shCTRL
3	1000	4	4	A549 shCTRL
4	10000	4	4	A549 shCTRL
5	10	6	0	A549 shLDHB-1
6	100	6	2	A549 shLDHB-1
7	1000	6	4	A549 shLDHB-1
8	10000	4	3	A549 shLDHB-1
9	10	6	0	A549 shLDHB-2
10	100	6	0	A549 shLDHB-2
11	1000	6	1	A549 shLDHB-2
12	10000	4	2	A549 shLDHB-2

k

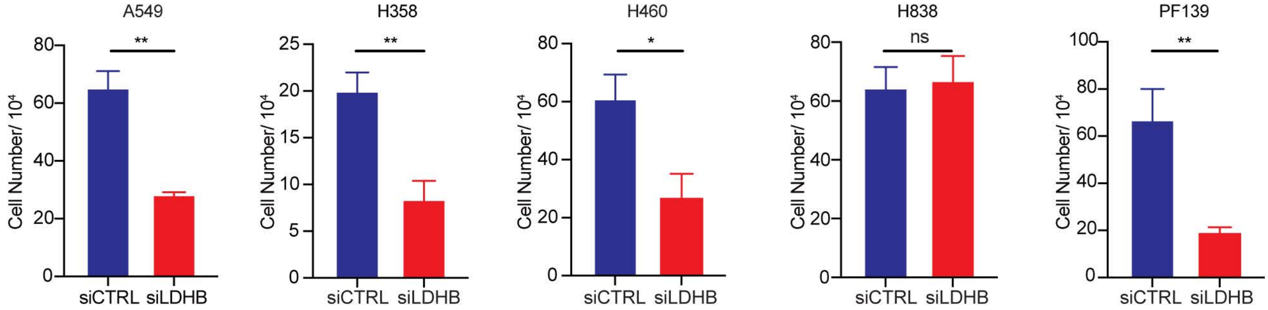

l

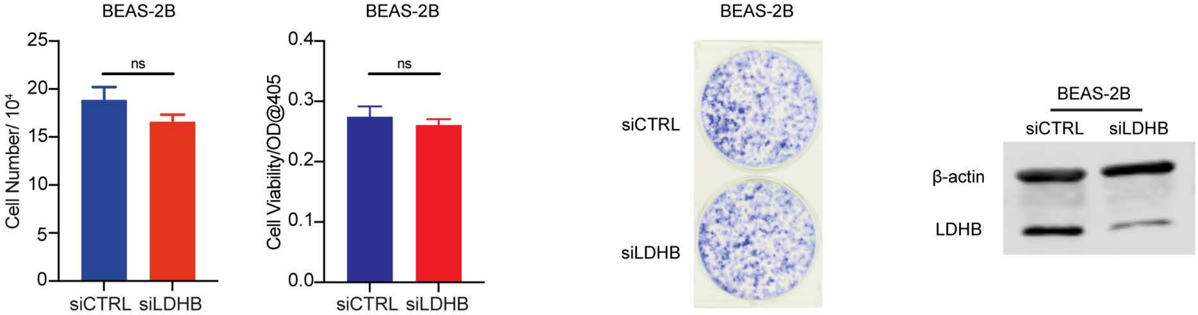

Fig. S4

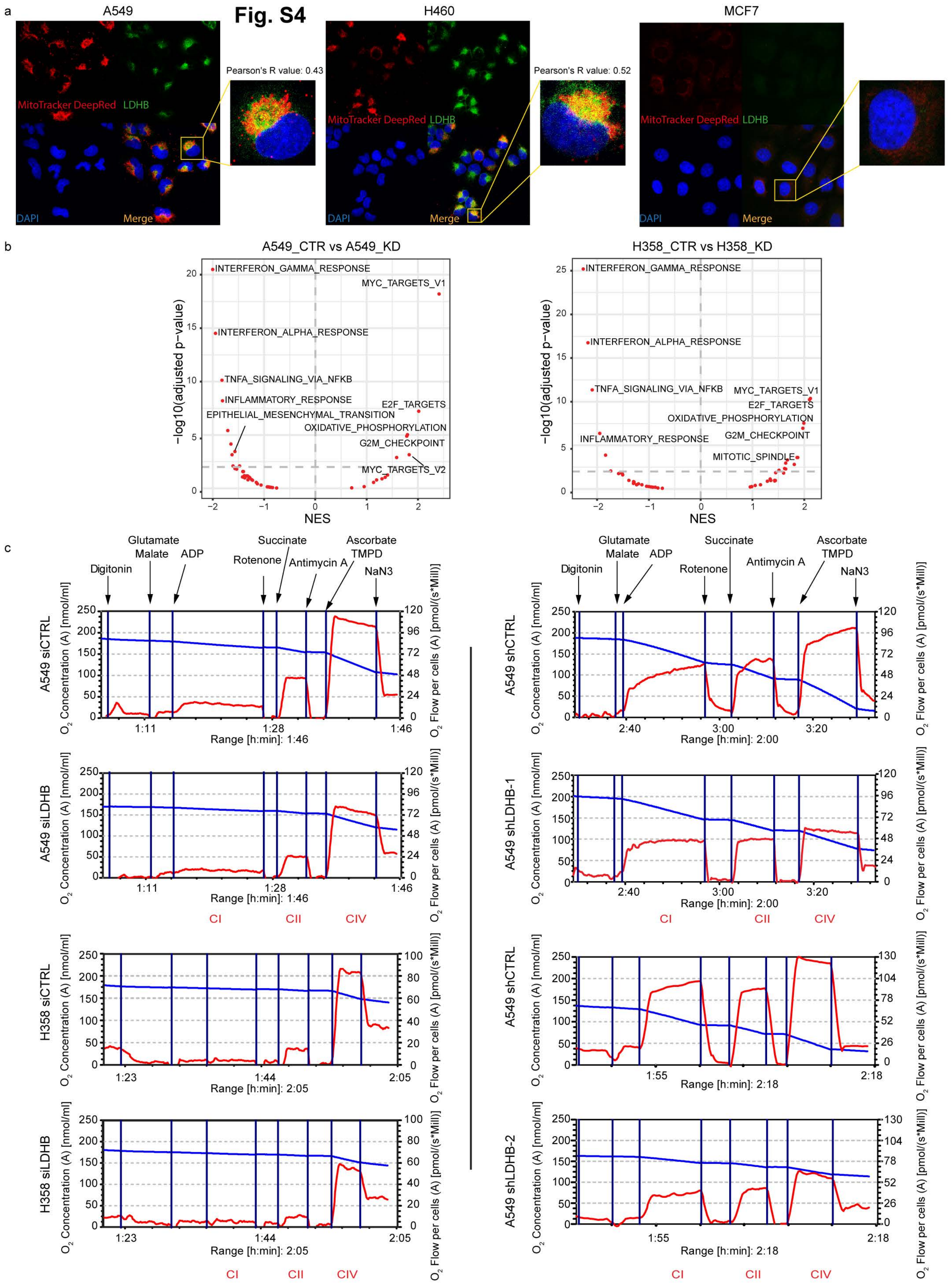

a
A549
Pearson's R value: 0.43
MitoTracker DeepRed
LDHB
DAPI
Merge
H460
Pearson's R value: 0.52
MitoTracker DeepRed
LDHB
DAPI
Merge
MCF7
MitoTracker DeepRed
LDHB
DAPI
Merge
b
A549_CTR vs A549_KD
INTERFERON_GAMMA_RESPONSE
MYC_TARGETS_V1
INTERFERON_ALPHA_RESPONSE
TNFA_SIGNALING_VIA_NFKB
INFLAMMATORY_RESPONSE
E2F_TARGETS
EPITHELIAL_MESENCHYMAL_TRANSITION
OXIDATIVE_PHOSPHORYLATION
G2M_CHECKPOINT
MYC_TARGETS_V2
−log10(adjusted p−value)
NES
H358_CTR vs H358_KD
INTERFERON_GAMMA_RESPONSE
INTERFERON_ALPHA_RESPONSE
TNFA_SIGNALING_VIA_NFKB
MYC_TARGETS_V1
E2F_TARGETS
OXIDATIVE_PHOSPHORYLATION
INFLAMMATORY_RESPONSE
G2M_CHECKPOINT
MITOTIC_SPINDLE
−log10(adjusted p−value)
NES
c
Digitonin
Glutamate
Malate
ADP
Rotenone
Succinate
Antimycin A
Ascorbate
TMPD
NaN3
A549 siCTRL
A549 siLDHB
H358 siCTRL
H358 siLDHB
A549 shCTRL
A549 shLDHB-1
A549 shCTRL
A549 shLDHB-2
O2 Concentration (A) [nmol/ml]
O2 Flow per cells (A) [pmol/(s*Mill)]
Range [h:min]: 1:46
Range [h:min]: 2:00
Range [h:min]: 2:05
Range [h:min]: 2:18
CI
CII
CIV

d

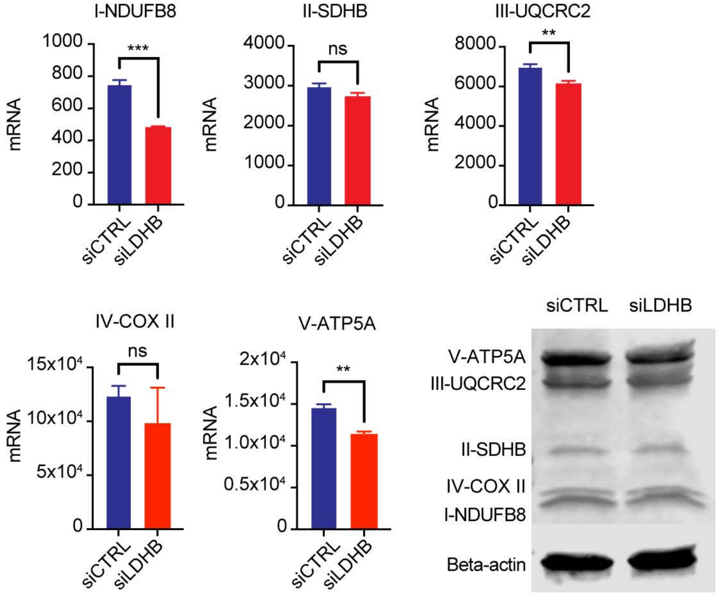
I-NDUFB8
II-SDHB
III-UQCRC2
mRNA
1000
800
600
400
200
0
***
4000
3000
2000
1000
ns
8000
6000
4000
2000
**
siCTRL
siLDHB
IV-COX II
V-ATP5A
15x10⁴
10x10⁴
5x10⁴
2x10⁴
1.5x10⁴
1.0x10⁴
0.5x10⁴
siCTRL
siLDHB
V-ATP5A
III-UQCRC2
II-SDHB
IV-COX II
I-NDUFB8
Beta-actin

e

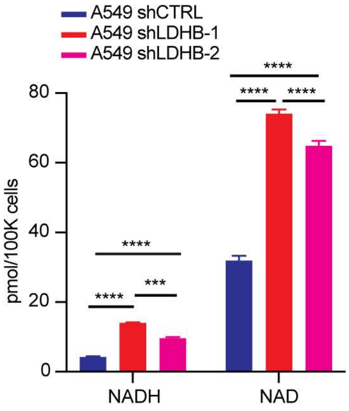
A549 shCTRL
A549 shLDHB-1
A549 shLDHB-2
pmol/100K cells
80
60
40
20
0
****
***
NADH
NAD

Fig. S5

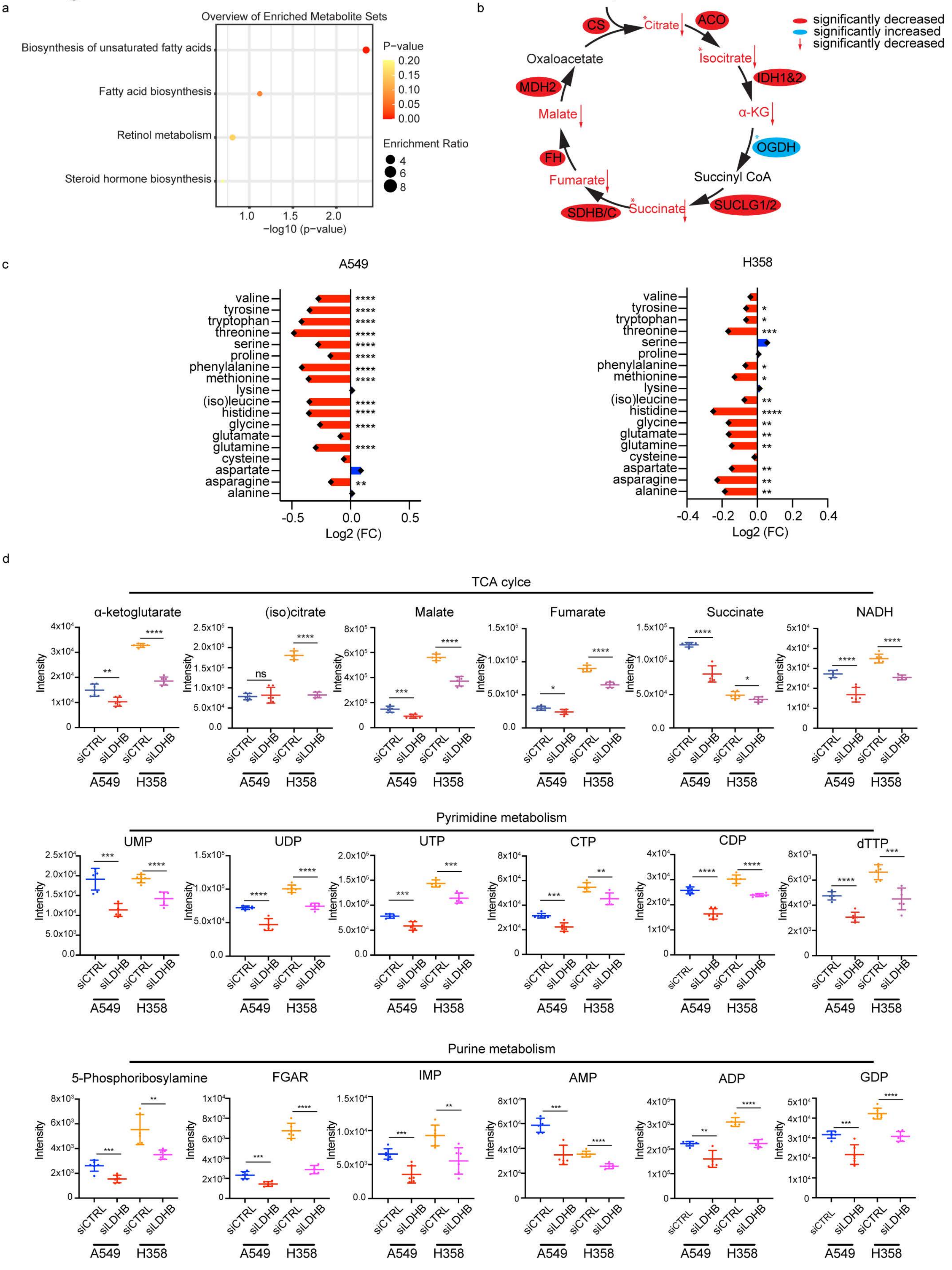
a
Overview of Enriched Metabolite Sets
Biosynthesis of unsaturated fatty acids
Fatty acid biosynthesis
Retinol metabolism
Steroid hormone biosynthesis
P−value
0.20
0.15
0.10
0.05
0.00
Enrichment Ratio
4
6
8
1.0
1.5
2.0
−log10 (p−value)
b
Acetyl CoA
CS
Citrate
ACO
Isocitrate
IDH1&2
α-KG
OGDH
Succinyl CoA
SUCLG1/2
Succinate
SDHB/C
Fumarate
FH
Malate
MDH2
Oxaloacetate
significantly decreased
significantly increased
significantly decreased
c
A549
H358
valine
tyrosine
tryptophan
threonine
serine
proline
phenylalanine
methionine
lysine
(iso)leucine
histidine
glycine
glutamate
glutamine
cysteine
aspartate
asparagine
alanine
Log2 (FC)
d
TCA cylce
α-ketoglutarate
(iso)citrate
Malate
Fumarate
Succinate
NADH
Pyrimidine metabolism
UMP
UDP
UTP
CTP
CDP
dTTP
Purine metabolism
5-Phosphoribosylamine
FGAR
IMP
AMP
ADP
GDP
Intensity
siCTRL
siLDHB
A549
H358
ns

Fig. S5

e

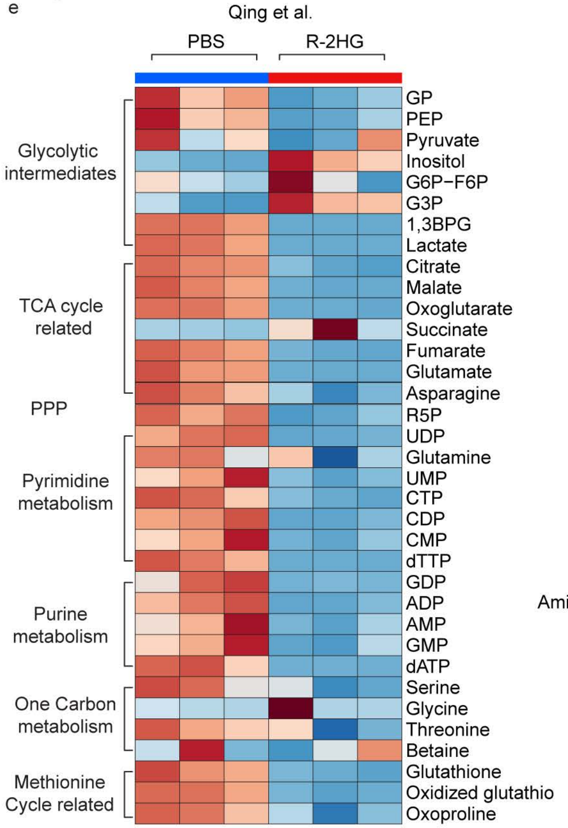

Qing et al.
PBS
R-2HG
Glycolytic intermediates
TCA cycle related
PPP
Pyrimidine metabolism
Purine metabolism
One Carbon metabolism
Methionine Cycle related
GP
PEP
Pyruvate
Inositol
G6P−F6P
G3P
1,3BPG
Lactate
Citrate
Malate
Oxoglutarate
Succinate
Fumarate
Glutamate
Asparagine
R5P
UDP
Glutamine
UMP
CTP
CDP
CMP
dTTP
GDP
ADP
AMP
GMP
dATP
Serine
Glycine
Threonine
Betaine
Glutathione
Oxidized glutathio
Oxoproline

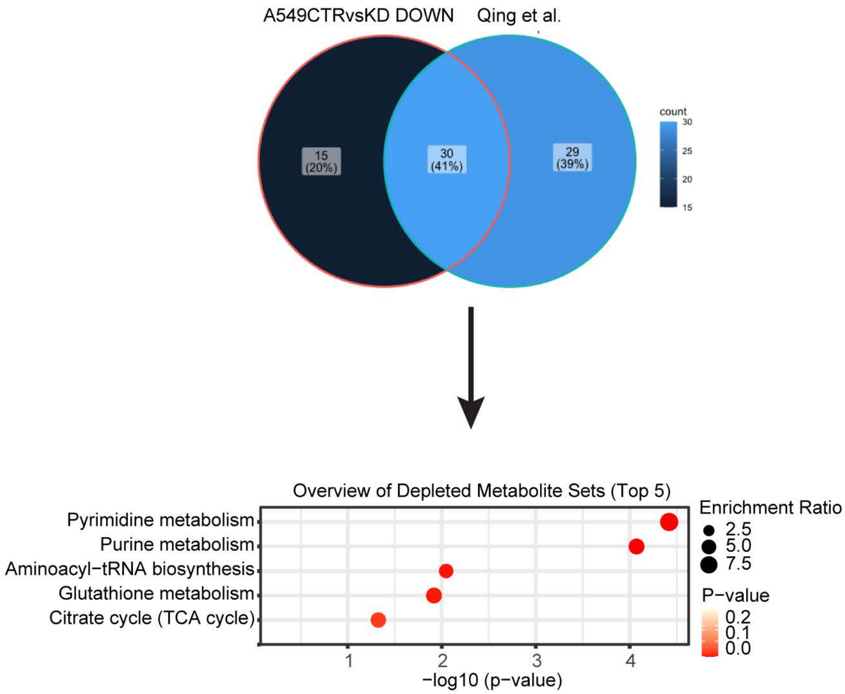

A549CTRvsKD DOWN
Qing et al.
15 (20%)
30 (41%)
29 (39%)
count
30
25
20
15
Overview of Depleted Metabolite Sets (Top 5)
Pyrimidine metabolism
Purine metabolism
Aminoacyl−tRNA biosynthesis
Glutathione metabolism
Citrate cycle (TCA cycle)
1
2
3
4
−log10 (p−value)
Enrichment Ratio
2.5
5.0
7.5
P−value
0.2
0.1
0.0

f

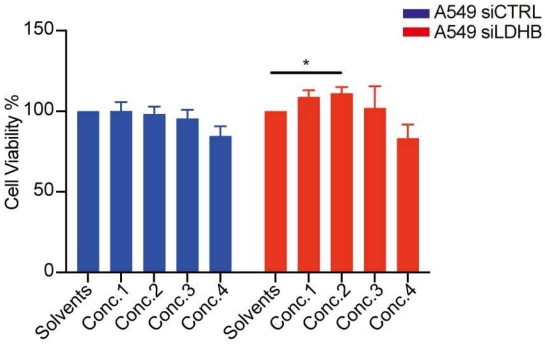

A549 siCTRL
A549 siLDHB
Cell Viability %
150
100
50
0
*
Solvents
Conc.1
Conc.2
Conc.3
Conc.4
Solvents
Conc.1
Conc.2
Conc.3
Conc.4

Fig. S6

a

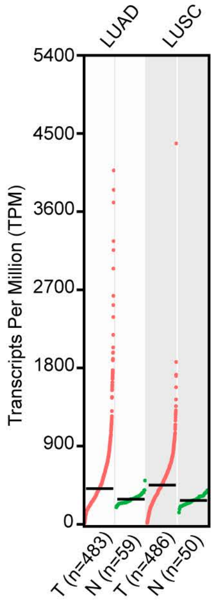

LUAD
LUSC
5400
4500
3600
2700
1800
900
0
Transcripts Per Million (TPM)
T (n=483)
N (n=59)
T (n=486)
N (n=50)

b

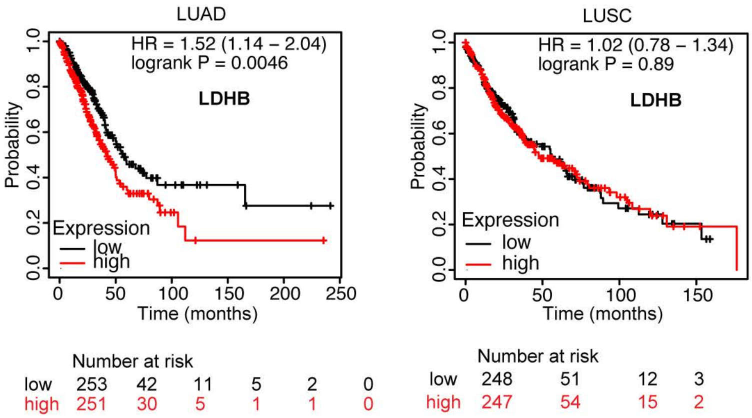

LUAD
HR = 1.52 (1.14 – 2.04)
logrank P = 0.0046
LDHB
Probability
Expression
low
high
Time (months)
Number at risk
low 253 42 11 5 2 0
high 251 30 5 1 1 0
LUSC
HR = 1.02 (0.78 – 1.34)
logrank P = 0.89
LDHB
Probability
Expression
low
high
Time (months)
Number at risk
low 248 51 12 3
high 247 54 15 2

c

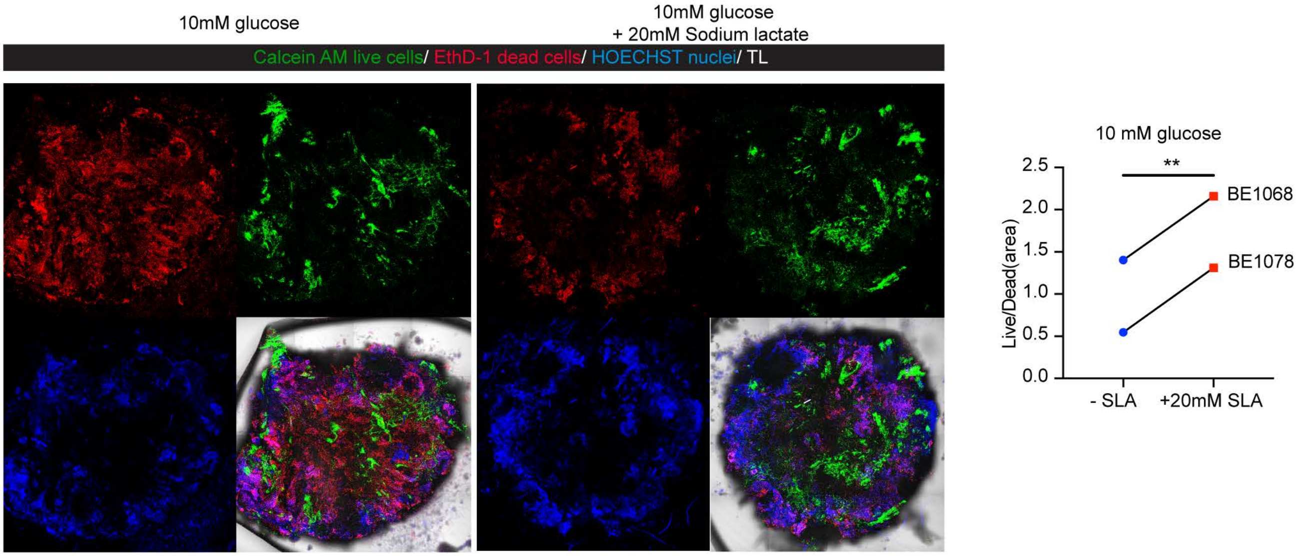

10mM glucose
10mM glucose
+ 20mM Sodium lactate
Calcein AM live cells/ EthD-1 dead cells/ HOECHST nuclei/ TL
10 mM glucose
**
BE1068
BE1078
Live/Dead(area)
- SLA
+20mM SLA

Fig. S7

a

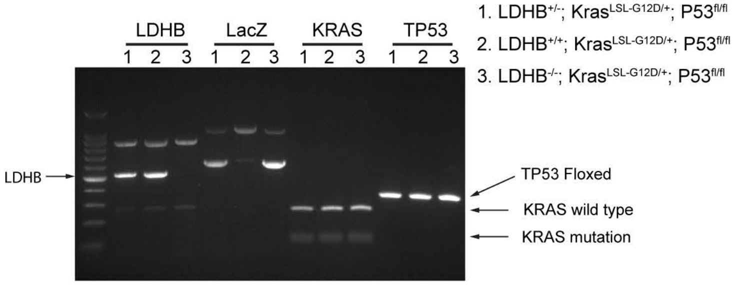

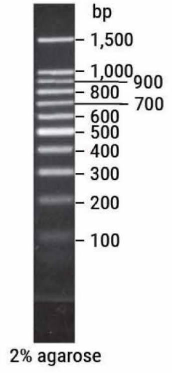

Marker	Size (bp)
LDHB	513
KRAS mut	100
KRAS wt	250
P53 Floxed	390
P53 WT	270
LacZ	608
VEGF	1038
Tie-1	856

b

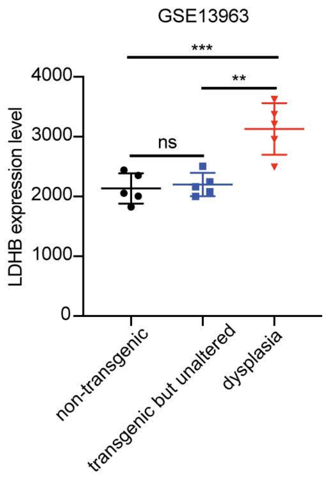

c

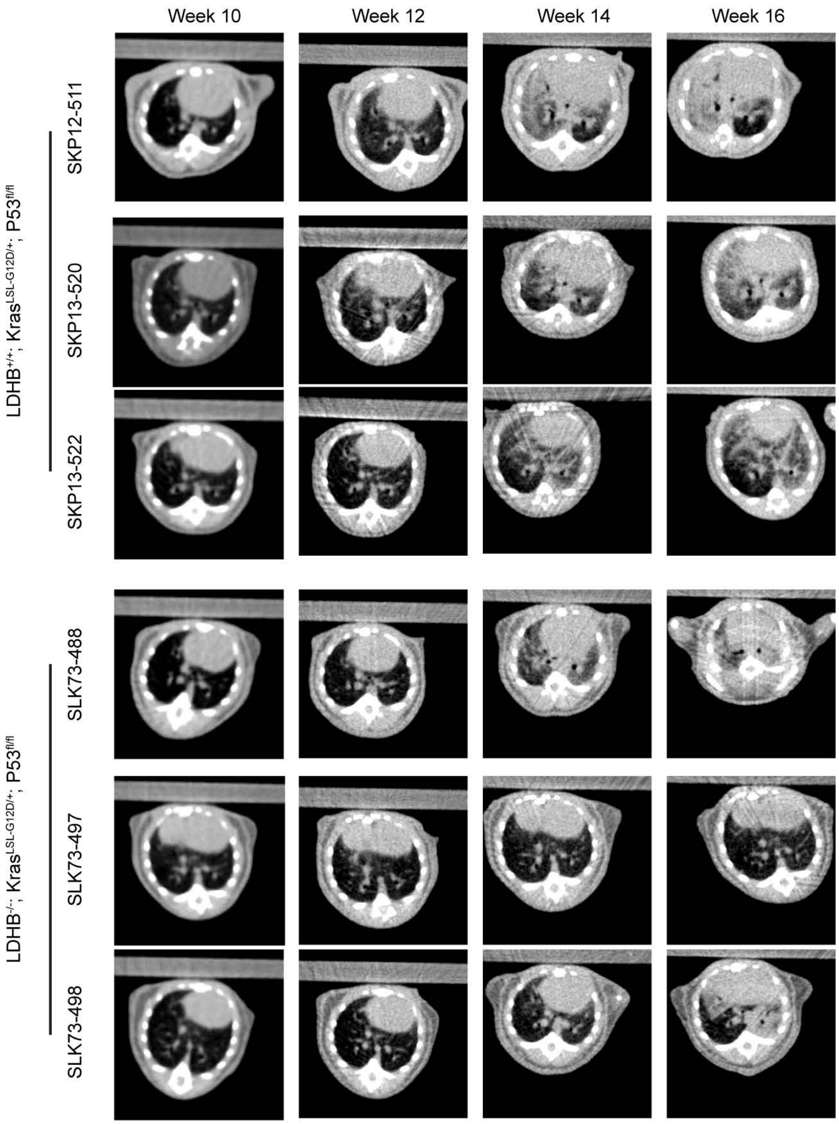

Supplement: Supplementary file 1 — Supplementary file1 Supplementary Fig. S1: a. Gene expression of selected genes in HOLO and PARA clones (n=3). *P < 0.05 (two-tailed unpaired Student’s t-test). b-c. Basal, ATP-linked, maximal, and spare oxygen consumption rate (OCR) of HOLO and PARA clones were measured as described in Fig.1. The OCR was normalized to mean OCR of HOLO cells. The error bars represent mean ± SD (n=3 biological replicates). ****P < 0.0001 (two-tailed unpaired Student’s t-test). d. The glycolytic function is represented by glycolysis, glycolytic capacity, and reserve glycolysis. The ECAR was normalized to mean ECAR of HOLO cells. The ECAR fold change to HOLO cells was shown by bar graph (n=4 biological replicates). The error bar was represented with mean ± SD. ***P < 0.001, ****P < 0.0001 (Ordinary two-way ANOVA). e. Flow cytometer plot with ALDEFLUOR (ALDH) or GLDC by using FlowJo for A549 and H358 cell lines, which were cultured in 20 mM L-lactic acid or HCl and adjusted to pH 6.8 as described in Fig.1. Supplementary Fig. S2: a. Immunoblot analysis of H358, H460, H838, and PF139 cells transfected with control siRNA (siCTRL) or LDHB-specific siRNA (siLDHB) (10 nM) using Lipofectamine 2000 after 48 hours. β-actin was used as the loading control. b-c. The oxygen consumption rate (OCR) of siCTRL and siLDHB cells were measured as described in Fig.1. The ATP-linked and maximal OCR of siLDHB cells were normalized to corresponding siCTRL and plotted as bar graphs (n=3-4 biological replicates). The error bars represent mean ± SD. ****P < 0.0001 (two-tailed unpaired Student’s t-test). d. Left panel: Representative plot showing mean ± SEM of the real-time extracellular acidification rate (ECAR) across treatments after 48 hours of transfection with siCTRL or siLDHB using the Seahorse XFe96 analyzer (n=23 technical replicates with 3-5 readings). Right panel: Extracellular acidification rate (ECAR) of siLDHB cells from Mito Stress Test was normalized to corresponding siCTRL and plotted as bar grap [file 18_2022_4453_MOESM1_ESM.pdf]
