# Supplementary material for: Targeting lactate dehydrogenase B-dependent mitochondrial metabolism affects tumor initiating cells and inhibits tumorigenesis of non-small cell lung cancer by inducing mtDNA damage
Source: Cell Mol Life Sci. 2022 Jul 25;79(8):445. doi: 10.1007/s00018-022-04453-5 (PMC9314287; doi:10.1007/s00018-022-04453-5)
Supplement: Supplementary file 2 — Supplementary file2 (PDF 139 KB) [file 18_2022_4453_MOESM2_ESM.pdf]

Table S1. Antibodies

Application	Antibody	Cat.#	Label	Dilution
Western Blot	LDHB	MAB9205-100; R&D Systems	No	1:20'000
	LDHA	3582S; Cell Signaling Technology	No	1:1'000
	beta-Actin (mouse)	3700S; Cell Signaling Technology	No	1:20'000
	beta-Actin (rabbit)	4970S; Cell Signaling Technology	No	1:1'000
	EpCAM	NB600-1182SS; Novus Biologicals	No	1:1'000
	Vimentin	5741; Cell Signaling Technology	No	1:1'000
	Total OXPHOS Human WB Antibody Cocktail	ab110411; abcam	No	1:500
Flow cytometer	EpCAM	25-9326-42; eBioscience	PE-Cyanine7	1:100
	EpCAM	50-9326-42; eBioscience	eFluor 660	1:100
	CD90	562385; BD Biosciences	CF549	1:100
	Anti-phospho Histone H2A.X^{Ser139}	05-636-AF488; EMD Millipore	Alexa Fluor® 488	1:100
	GLDC	bs-13370R-A555; Bioss Antibodies	Alexa Fluor® 555	1:100
	SOX2	53-9811-82; Thermo Fisher Scientific	Alexa Fluor® 488	1:100
	SOX2	130-104-994; Miltenyi Biotec	PE	1:100
	LDHB	IC9205S; R&D Systems	Alexa Fluor® 750	1:100

Immunofluorescence	LDHB	IC9205G; R&D Systems	Alexa Fluor® 488	1:100
	LDHB	IC9205R; R&D Systems	Alexa Fluor® 647	1:100
	LDHA	sc-137243; Santa Cruz Biotechnology	Alexa Fluor® 546	1:100
Immunohistochemistry	LDHB	MAB9205-100; R&D Systems	No	1:40’000
	GLDC	HPA002318; Sigma	No	1:200

Table S2. Chemicals

Name	Company	Catalog Number
Glucose	Sigma-Aldrich	G7021
L-glutamine	Thermo Fisher Scientific	25030024
Sodium pyruvate	Thermo Fisher Scientific	11360039
FCCP	Selleck Chemicals	S8276
Oligomycin	Sigma-Aldrich	O4876
Rotenone	Sigma-Aldrich	R8875
Antimycin A	Sigma-Aldrich	A8674
ADP	Sigma-Aldrich	A4386
Ascorbate	Merck	1.00127
BSA	Cell Signaling Technology	9998S
Digitonin	Sigma-Aldrich	D5628
EGTA	Sigma-Aldrich	E3889
Glutamate	Sigma	G1626
Hepes	Sigma	H7523
KCl	Merck	1.04936
KH2PO4	Merck	1.04873
K-lactobionate	Sigma	L2398
MgCl2	Sigma	M9272
Sodium azide	Sigma	S2002
Succinate	Sigma	S2378
TMPD	Sigma	T3134

Malate	Sigma-Aldrich	M7397-25G
Ammonium carbonate	Sigma-Aldrich	207861-25G
Acetonitrile	Sigma-Aldrich	34998-1L
Acetic acid		
Methanol		

Table S3 Primers

	Primer/probe from Microsynth		
**#**	**Labeled tube**	**Description**	**Seq**
**Ms156**	156 B-g taq-s	B-globin (from 13.5kb fragment) small fragment 258bp sense	GGCTGTCTCCTAGCAACGAC
**Ms157**	157 B-g taq-as	B-globin (from 13.5kb fragment) small fragment 258bp antisense	TGCATACCAGCTCTCACCTG
**Ms186**	186 Mito 221bp s	Mito 221bp sense (Short range mitoDNA)	CCC CAC AAA CCC CAT TAC TAA ACC CA
**Ms187**	187 Mito 221bp as	Mito 221bp antisense (Short range mitoDNA)	TTT CAT CAT GCG GAG ATG TTG GAT GG
**Ms30**	30 5'B-globin s	5'B-globin 13.5kb sense	CGA GTA AGA GAC CAT TGT GGC AG
**Ms31**	31 5'B-globin as	5'B-globin 13.5kb antisense	GCA CTG GCT TAG GAG TTG GAC T
**Ms32**	32 mito 16.2kb s	mito 8.9kb sense (Long range mitoDNA)	TCT AAG CCT CCT TAT TCG AGC CGA
**Ms33**	33 mito 16.2kb as	mito 8.9kb antisense (Long range mitoDNA)	TTT CAT CAT GCG GAG ATG TTG GAT GG
**Ms34**	34 hprt6 10.4kb s	hprt6 10.4k sense	TGG GAT TAC ACG TGT GAA CCA ACC
**Ms35**	35 hprt6 10.4k as	hprt6 10.4k antisense	GCT CTA CCC TCT CCT CTA CCG TCC
